# Efficacy of Hair Total Mercury Content as a Biomarker of Methylmercury Exposure to Communities in the Area of Artisanal and Small-Scale Gold Mining in Madre de Dios, Peru

**DOI:** 10.3390/ijerph182413350

**Published:** 2021-12-18

**Authors:** Faye Koenigsmark, Caren Weinhouse, Axel J. Berky, Ana Maria Morales, Ernesto J. Ortiz, Eric M. Pierce, William K. Pan, Heileen Hsu-Kim

**Affiliations:** 1 Civil and Environmental Engineering, Duke University, Durham, NC 27708, USA; faye.koenigsmark@duke.edu; 2 Oregon Institute of Occupational Health Sciences, Oregon Health & Science University, 3181 SW Sam Jackson Park Road, Portland, OR 97239, USA; weinhous@ohsu.edu; 3 Nicholas School of the Environment, Duke University, 9 Circuit Drive, Durham, NC 27710, USA; axel.berky@duke.edu; 4 Centro Nacional de Salud Intercultural, Instituto Nacional de Salud, Ministerio de Salud, Cápac Yupanqui 1400-Jesus María, Lima 15027, Peru; anamorales30@hotmail.com; 5 Duke Global Health Innovation Center, Duke University, 310 Blackwell Street, Durham, NC 27701, USA; ernesto.ortiz@duke.edu; 6 Environmental Sciences Division, Oak Ridge National Laboratory, 1 Bethel Valley, Oak Ridge, TN 37831, USA; pierceem@ornl.gov; 7 Duke Global Health Institute, Duke University, 310 Trent Drive, Durham, NC 27710, USA

**Keywords:** mercury, exposure biomarker, population monitoring, mining

## Abstract

Total mercury content (THg) in hair is an accepted biomarker for chronic dietary methylmercury (MeHg) exposure. In artisanal and small-scale gold mining (ASGM) communities, the validity of this biomarker is questioned because of the potential for contamination from inorganic mercury. As mining communities may have both inorganic and organic mercury exposures, the efficacy of the hair-THg biomarker needs to be evaluated, particularly as nations begin population exposure assessments under their commitments to the Minamata Convention. We sought to validate the efficacy of hair THg for public health monitoring of MeHg exposures for populations living in ASGM communities. We quantified both THg and MeHg contents in hair from a representative subset of participants (N = 287) in a large, population-level mercury exposure assessment in the ASGM region in Madre de Dios (MDD), Peru. We compared population MeHg-THg correlations and %MeHg values with demographic variables including community location, sex, occupation, and nativity. We observed that hair MeHg-THg correlations were high (r > 0.7) for all communities, regardless of location or nativity. Specifically, for individuals within ASGM communities, 81% (121 of 150 total) had hair THg predominantly in the form of MeHg (i.e., >66% of THg) and reflective of dietary exposure to mercury. Furthermore, for individuals with hair THg exceeding the U.S. EPA threshold (1.0 μg/g), 88 out of 106 (83%) had MeHg as the predominant form. As a result, had urine THg solely been used for mercury exposure monitoring, approximately 59% of the ASGM population would have been misclassified as having low mercury exposure. Our results support the use of hair THg for monitoring of MeHg exposure of populations in ASGM settings where alternative biomarkers of MeHg exposure are not feasible.

## 1. Introduction

The Minamata Convention on Mercury is an international effort to reduce the negative impacts of anthropogenic mercury pollution. As part of global advancements towards this goal, signatory nations are establishing regional biomonitoring programs for mercury exposures. For populations near artisanal and small-scale gold mining (ASGM), biomonitoring for Hg exposure is a special challenge because individuals within these populations can be exposed to multiple forms of mercury, requiring different strategies for detection.

ASGM is a largely unregulated mining technique responsible for over 30% of anthropogenic mercury (Hg) emissions to the atmosphere [1], and involves the use of liquid elemental mercury (Hg^0^_L_) to amalgamate and separate gold from excavated soils [2]. The gold is refined by heating the amalgam and evaporating the mercury, a process occurring both at excavation sites as well as in gold shops at local population centers.

Individuals engaged in smelting and amalgamation activities in ASGM are exposed to inorganic Hg forms (e.g., Hg^0^_L_ vapors), which can be monitored through the analysis of urine biomarkers [3,4]. However, in mining areas where aquatic wildlife is an important protein source, such as ASGM regions throughout South America [5,6,7], Africa [8,9], and South Asia [10,11], methylmercury (MeHg) exposure is also a concern [5,12], particularly for children who are at risk for neurological impairment [13,14].

Chronic MeHg exposure cannot be evaluated by urine biomarkers [4]; rather this type of exposure is often evaluated by quantifying total mercury (THg) content in hair, a well-established biomarker of MeHg burden in the brain—its primary target tissue [15,16]. This biomarker relies on the assumption that the majority of THg in the matrix is MeHg, and with hair growth rate of approximate 1 cm per month, the biomarker represents multiple months of exposure, depending of the hair length analyzed [13,17,18,19]. For regions that host ASGM activity, however, the THg hair biomarker for population monitoring of MeHg exposure has not been recommended in the draft effectiveness evaluation of the Minamata Convention [4,20], due to questions raised in recent reports [13,21,22,23]. These doubts are based on studies of individuals engaged in ASGM activity showing that the percentage of hair THg as MeHg ranged from 1% to 110% (Supplemental Information Table S1) [22,24,25,26,27,28,29]. In contrast, control groups consisting of individuals from areas with no known ASGM activity had average hair %MeHg values of 84% ± 18% (Supplemental Information Table S2) [22,30,31,32,33,34,35,36,37]. Inorganic mercury (iHg) can accumulate in hair exogenously via deposition [38,39], as well as endogenously through demethylation of hair MeHg to iHg and absorption of ingested iHg and inhaled Hg^0^ vapors that are then incorporated into the growing hair [17,40,41]. The extent and rates of endogenous iHg accumulation processes are generally low relative to MeHg uptake in hair [13,17,42].

While these prior studies in ASGM areas suggest that hair THg levels could overestimate dietary MeHg exposure in the community [43], these studies are not designed to inform population monitoring practices as they used non-random selection of small, convenience samples with limited spatial variability, or they directly targeted individuals involved in gold refining [29]. For example, 5 of the 7 studies had N < 28 participants across 1–3 locations (Table S1). A relatively larger study (N = 128, 6 communities) entailed a case-control design in which cases were non-randomly selected individuals living in mining communities who were involved in smelting and controls were non-mining individuals living in non-mining communities. None of these study designs allow for understanding of the hair THg biomarker for mining communities where the residents include those not directly engaged in mining or smelting activities.

While extremely elevated gaseous Hg levels can occur in areas directly outside the gold shops of mining communities [44], the impact of these transient air quality events on inorganic Hg exposure to the overall population is unknown. Regardless, national biomonitoring programs such as Peru, Colombia, and Uganda exclude hair THg in their population surveillance plans for communities near ASGM due to uncertainties raised in reports that directly studied individuals engaged in amalgam burning. Instead, these programs recommend urine, an accepted biomarker for iHg exposure, and possibly THg in blood—a biomarker for recent MeHg and iHg exposure and not necessarily long-term exposure. Such policies could fail to identify chronic MeHg exposure risk within populations near ASGM areas. Population-relevant evaluations of the hair THg biomarker in ASGM areas are needed to validate the efficacy of this biomarker and to help inform best practices.

**Figure 1:**
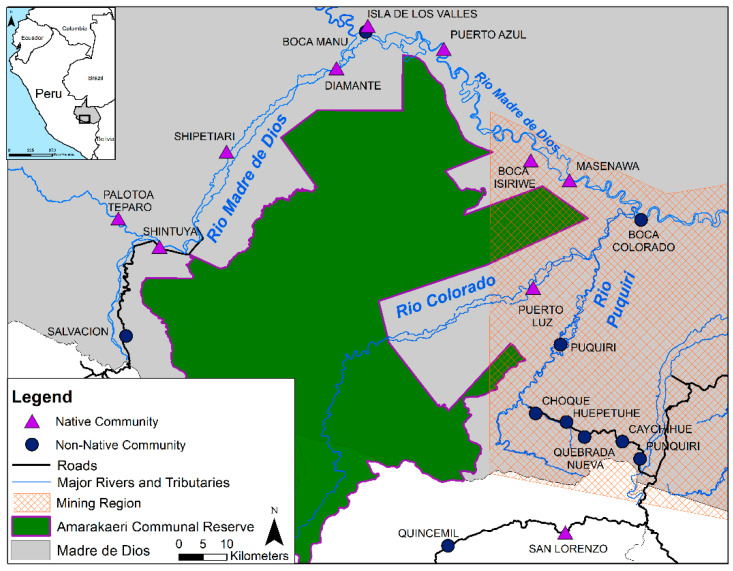
Map of the study area in MDD, Peru. Communities along the river were distinguished based on residence within ASGM (orange hashed zone) or outside ASGM (grey and white zones). Households were visited between 2015 and 2016 for collection of hair samples.

This study seeks to understand the efficacy of hair-THg as a biomarker of MeHg exposure in population biomonitoring of ASGM regions. To this end, we quantified the relationship between MeHg and THg contents in hair for individuals in Madre de Dios, Peru (Figure 1), a region that is an epicenter of ASGM activities in the Amazon basin over the last two decades and has experienced mercury releases to the river environment [45,46,47,48,49,50,51]. Mercury exposures to levels that exceed health guidelines have been widely documented for residents of the region [5,12,52,53,54,55,56]. We quantified THg and MeHg contents in hair obtained from N = 287 individuals (of N = 2167 sampled) from 20 communities located within and near the mining region. Approximately half of these individuals were living in ASGM communities where Hg exposures could be both MeHg and iHg, and the other half were living in non-mining communities where mercury exposures are presumed to be primarily MeHg through diet [5,12,52,57]. Single timepoints of exposure as well as temporal, chronic trends were evaluated from communities representing a spectrum of geographic locations and diet, including both native, fish-consuming communities and mining towns populated by migrants from regions where multiple types of dietary protein are consumed.

## 2. Materials and Methods

### 2.1. Study Design and Sample Collection

#### 2.1.1. Population Cohort

This study is part of our larger parent study [52,53] to evaluate the health status of 23 communities located around the Amarakaeri Communal Reserve along the Madre de Dios and Puquiri Rivers in Southeastern Peru (Figure 1). During March to June 2015, our research team visited 1221 households to collect hair and blood samples, and administer household surveys that included a module on dietary intake from individuals [53]. Households were enrolled if they contained at least one woman of childbearing age (WCBA, 15–49 years). For every 9 households enrolled using this inclusion criteria, a 10th was enrolled without that requirement. All members of enrolled households were offered to receive hair THg testing. Within each household, a “sentinel group” was defined consisting of a WCBA and, if applicable, her spouse and her child. More than one sentinel group could be defined within a household depending on household structure. Adults classified in the sentinel group were offered additional mercury blood testing [52,53]. Data collection included a household survey, administered to the economic head of the household, which included questions of individual demographics (age, sex, socioeconomic position), and household food sources. Occupation was initially a 17-category variable in the survey. We later grouped the responses into six categories for ease of computation: mining, agriculture/fishing, other outdoor (e.g., logger, hired labor), professional/urban (e.g., teacher), stay at home/other, and unemployed.

Hair samples were collected from individuals by cutting three tufts of hair from different parts of the occipital region of the scalp. Each tuft was attached to self-adhesive note paper and stored separately in paper envelopes. All samples were stored at ambient conditions and transported to Duke University for analysis.

As noted in our prior publication for the parent study [53], this study was approved by the Committee on Human Ethics from the Universidad Peruana Cayetano Heredia (UPCH) (OHRP registration IORG0000671, IRB00001014, study ID #63056).

#### 2.1.2. Subsample Cohort

This current study is a subset of our parent study: individuals 18 years and older and part of sentinel households. Individuals were selected for MeHg analysis if they: (a) had blood Hg measurements; (b) had blood polyunsaturated fatty acid measurements (PUFAs, a biomarker possibly linked to diet); and (c) had two remaining intact tufts of hair (of the original three tufts taken). In total, the hair samples of N = 287 individuals across 20 communities were selected for Hg speciation analysis for this study. For data analysis, communities were classified into two distinct geographical regions: within ASGM activity (N = 150 study participants from 10 communities), and outside of ASGM (N = 137 study participants from 10 communities). Community designations as ‘within’ or ‘outside’ of mining were defined by the Amazon Conservation Association (Asociación para la Conservación de la Cuenca Amazónica, ACCA). Descriptive statistics of individuals for this study in each region are presented in Table 1 and Table S5. Individuals from indigenous communities and non-indigenous communities were also compared, with indigenous communities shown to be more reliant on fish consumption. Indigenous, or native, communities were defined as those listed on Peru’s Ministry of Culture database of indigenous populations at http://bdpi.cultura.gob.pe (Accessed on 1 August 2019). Among the 20 communities represented in this study, 10 were native. Most of the native communities are located outside of mining activity while three are located within mining.

In a subset of 23 individuals, we assessed temporal MeHg exposure via distal hair segment analysis (Section 2.3). We designated non-migratory status to certain individuals if the person lived in the same community for at least five years, had traveled outside their community less than five days in the last three months, and had no occupational labor outside the community.

### 2.2. Mercury Analyses in Hair Specimens

Of the three hair tufts collected from each individual, one was analyzed for THg content, another tuft analyzed for MeHg, and the third was saved for potential future analyses. Both measurements were performed on the proximal 2 cm segments of each hair tuft (unwashed), which corresponds to exposure over the most recent two-month period before sample collection [23]. We chose not to wash the hair samples because washing procedures have been reported to leach mercury compounds incorporated into the hair at the follicle, resulting in underestimation of hair mercury content [58,59,60,61,62,63].

THg data for proximal segments from this population were reported by Weinhouse et al. [53]. For all other segments processed for this study, THg content was determined using the same methods, by thermal decomposition, amalgamation, and atomic absorption detection (Milestone DMA-80). The instrument was calibrated with dilutions of a certified 1 mg/L Methylmercury Standard (Brooks Applied Labs, Bothell, WA, USA). Calibration was verified by running a hair certified reference material (CRM DB001, European Reference Materials) after every 10 to 15 samples. Sample measurements were accepted if the subsequent analysis of the CRM was within 10% of the certified mean value. The average recovery of the THg reference value in the CRM was 98% ± 6.9% (N = 308) (SI Section S2, Figure S1). Additional information on the uncertainty of THg measurements is provided in the SI Section S3.

MeHg content was determined by extraction with tetramethylammonium hydroxide [21], followed by analysis of extracts by aqueous phase ethylation, purge-trap on Tenax resin and gas chromatographic separation (BrooksRand MerxM), and inductively coupled plasma mass spectrometry (Agilent 7700). Duplicate extractions of hair reference material (RM; International Atomic Energy Agency 086) were performed in parallel with each batch of samples. An aliquot of stable MeHg isotope (CH_3_^201^HgCl) was added as an internal standard (IS) to each RM and hair sample prior to the extraction step. Measured MeHg values of the RM were 99% ± 8.4% (N = 50) of the reference value, after adjustment of the measured IS (Figure S2B). For the hair samples, the average IS recovery was 82% ± 14% for proximal hair segments and 93% ± 18% for distal hair segments (Figure S4). Additional descriptions and results of instrument calibration verification, IS recoveries, RM analyses, and measurement uncertainty are presented in SI Sections S2 and S3.

### 2.3. Intra-Individual Variation Evaluated with Distal Hair Segment Analyses

**Figure 2:**
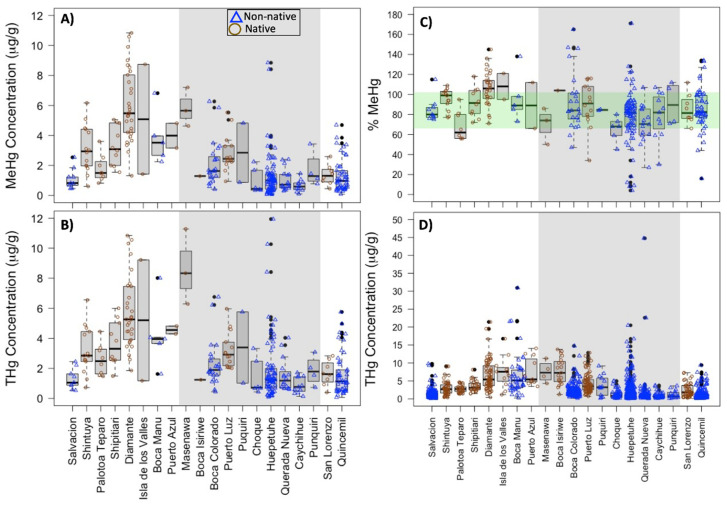
(**A**) MeHg, (**B**) THg, and (**C**) the % of THg as MeHg in the hair for individuals (N = 287) living in communities around the Amaerakari Reserves in Madre de Dios, Peru. These individuals are a subset from the larger parent study (N = 1543) [53] with hair THg distribution shown in (**D**). Measurements were performed on the proximal 2-cm hair segment. Symbols represent measurements of individual participants and are grouped by their residence in native (open brown circles) and non-native (open blue triangles) communities. Communities in the grey shaded region lie within mining. In part (**C**), the light green shaded region represents the expected range of %MeHg values, based on prior studies of Hg exposure for individual exposed to mercury only through diet. The black bars represent the median hair concentrations for each community, the box outline represents values in the 25th to 75th percentile, the whiskers represent values outside the middle 50%, and the closed black circles represent data points that are outliers of each community distribution.

After analysis, we observed that for several samples, the measured MeHg relative to measured THg (expressed as %MeHg) was over 100% (Figure 2). We hypothesized that this was due to analyzing MeHg and THg on separate tufts of hair. Note that variation of hair mercury content is expected on different parts of an individual’s scalp, which may contribute to intra-individual differences in the data. We therefore performed an additional analysis for selected individuals with long hair samples (from female individuals) in which the two tufts were combined into a single composite sample that was then cut into 2-cm segments along the entire length of hair. These 2-cm segments were then apportioned for analysis of MeHg and THg using the methods described above.

Distal hair segment analyses were performed for N = 23 individuals from two communities: the mining community Huepetuhe (HHU) (N = 15) and the non-mining, native community Diamante (HDM) (N = 8). The individuals within each community were selected to obtain a spectrum of proximal %MeHg values grouped into three categories: %MeHg < 66% (N = 6); 66% ≤ %MeHg ≤ 102% (N = 9); %MeHg > 102% (N = 8), which correspond to %MeHg values below, within, and above the %MeHg reference range, respectively (See Reference Hair %MeHg Range in Section 2.4.1 for explanation of these thresholds). THg and MeHg contents in composited distal hair segments were performed to understand potential discrepancies introduced in the analysis of non-composite proximal segments for THg and MeHg contents.

### 2.4. Statistical Analyses

Descriptive statistics with the median, range, and mean were used to describe the study population. Significant differences in hair THg, MeHg, and %MeHg between community types (native vs. non-native and within vs. outside of mining) were evaluated with either a Student or Welch two-sample *t*-test at the level α < 0.95. Hair THg was compared to the U.S. Environmental Protection Agency’s (EPA) reference level of 1.0 μg/g [19]. The proportion of individuals exceeding this limit was reported by native/non-native, as well as location relative to mining region (i.e., within versus outside).

#### 2.4.1. Reference Hair %MeHg Range

To compare the accuracy of hair THg as a predictor for MeHg in these dual exposure communities, the %MeHg values were compared to values expected for a control population exposed solely to MeHg through diet. The pooled mean ± standard deviation of hair %MeHg measurements was 84% ± 18% for fish-consuming populations in Belgium [30], Brazil [26,31,35], Yugoslavia [32], Iraq [33], Spain [34,36], and France [22] (Table S2). Thus, we grouped individuals into categories within or outside the range of 66–102% for %MeHg (i.e., the “expected” range if individuals were only exposed to mercury through fish consumption).

#### 2.4.2. Predictors of Hair THg, Hair MeHg, and %MeHg

Household survey data for sex, age, occupation, nativity, and location relative to mining were treated as categorical variables (SI Section S5, Table S6). We first tested bivariate associations between each predictor variable and outcomes hair THg, hair MeHg, and %MeHg (Tables S7–S9). Variables with associations of *p* < 0.20 were included in a multivariate linear regression model. Random intercepts were used to control for correlation within communities. The distributions of all outcome variables were right skewed; thus, natural log transformation was used in all analyses.

Local join count tests were performed in GeoDa (v1.18) to test for spatial clustering of individuals in mining regions with %MeHg values above and below the expected threshold. Logistic regressions were also performed to understand other variables associated with these individuals. We calculated the odds ratio and probability that an individual exhibits hair %MeHg < 66% for each of the predictor variables described above. Random intercepts were used to control for correlation within communities. Predictor variables were included in the final multivariate analysis if the bivariate association tested had a *p* < 0.20.

#### 2.4.3. Hair THg-Hair MeHg Correlations

Pearson correlations were computed between natural log-transformed hair THg and natural log-transformed hair MeHg values. Correlations were performed for all individuals as well as for individuals grouped by nativity, location relative to mining, and community.

## 3. Results

### 3.1. Differences in Hair THg, MeHg, and %MeHg Contents between Communities

For the subsample selected for this study, the median THg value in the proximal 2-cm hair segments was 1.51 μg/g (range 0.090–11.9 μg/g) and 2.55 μg/g (range 0.070–10.8 μg/g) for residents living within (N = 150) and outside (N = 137) of mining communities, respectively (Table 1, Figure 2B).These THg values correspond to 71% and 80% of the residents living within and outside of mining surpassing the US EPA threshold value of 1.0 μg/g hair THg, respectively. Median hair MeHg contents were 1.14 μg/g (range 0.059–8.85 μg/g) and 1.97 μg/g (range 0.080–10.8 μg/g) for individuals living within and outside mining, respectively (Table 1, Figure 2A). We observed the median percentage of hair THg as MeHg (%MeHg) was 82% (range 4–171%) for individuals within mining and 91% (range 16–145%) for individuals outside of mining (Table 1, Figure 2C).

While %MeHg values are theoretically bound between 0 and 100%, subsequent sensitivity analysis attributed %MeHg values over 100% to intra-individual variation in hair samples (see SI Sections S3 and S4). For eight individuals with %MeHg values of 105–170% in non-homogenized proximal hair segments, the adjacent homogenized distal segment had %MeHg values in the expected range (66–103% MeHg) (Figure S6). In contrast, five out of six individuals with %MeHg below 66% in the proximal segment also had <66%MeHg in the homogenized distal segment. Variation of THg content across three different hair tufts appeared to explain %MeHg values exceeding the 66–102% expected range.

The individuals living near mining activity may have experienced mercury exposures from both inorganic and organic Hg sources. Regardless, median %MeHg values for all communities are within the expected range (66% to 102% shown in the green shaded area of Figure 2C) based on populations with dietary MeHg as the sole exposure source (Table S2) [22,64]. Using >66%MeHg as a cutoff, we observed that 127 out of 137 people outside of mining (92.7%) and 121 out of 150 people within mining (80.7%) had MeHg as the predominant form of THg in their hair (Table 1).

### 3.2. Other Demographic Variables Associated with Exposure

To further assess the efficacy of hair THg as a predictor for MeHg exposure, mixed effect models were used to evaluate if other demographic variables were correlated with THg, MeHg, and %MeHg values. Residence in a native community was associated with 2.18 (95% CI: 1.38–3.35) and 2.22 (95% CI: 1.23–3.67) times higher hair THg and MeHg content than non-native communities, respectively (Tables S7 and S8). The significance of nativity in both the parent study [53] and in this subset population further support that consumption of contaminated fish (potentially from ASGM-related mercury discharge) is a main source of exposure for indigenous individuals.

Residence within/outside of mining, age, and occupation were not significantly associated with hair MeHg (Table S8, Figure S9) or hair THg (Table S7, Figure S8) at the 0.05 level. However, sex was a significant factor associated with THg in hair, predicting 24% lower hair THg levels in females compared to males (95% CI: 6.3–29.3%).

Importantly, correlations between %MeHg and demographic variables could inform efficacy of the THg hair biomarker for MeHg exposure. Given our observations of higher hair MeHg levels in native communities, residence in a non-native community within mining was hypothesized to contribute to a lower %MeHg due to potential for inorganic Hg exposure in these areas. However, native ethnicity was not a significant predictor of %MeHg. In fact, none of the variables included in the multivariate analysis for %MeHg (nativity, residence within mining, and occupation) were significant at the 0.05 level (Table S9, Figure S10). Residence within mining was a significant predictor for %MeHg (*p* < 0.05) in the bivariate analysis but was not significant in the multivariate model.

### 3.3. Factors Associated with Low %MeHg Values in Hair in Mining Communities

Spatial analyses and logistic regressions were performed to understand factors that may differentiate individuals within mining communities whose %MeHg values were above and below the 66% threshold. The demographics of these two subsets are presented in Table 2, and both groups demonstrated comparable characteristics. We hypothesized that, for each community, individuals with less than 66%MeHg may be clustered geographically (i.e., located near a gold smelting shop). Although we could not test spatial clustering of MeHg distributions as a function of gold shop locations since these locations were not available, join count statistics indicated no significant clustering of individuals living in Huepetuhe, Caychihue, or Boca Colorado (*p* > 0.05).

Logistic regressions, however, indicated that sex and certain occupations were associated with individuals exhibiting %MeHg less than 66% (Table S10, Figures S11 and S12). Females had 0.25-times lower odds of having %MeHg < 66% than males. This is consistent with the fact that males made up 41% of individuals with %MeHg < 66%, despite representing only 30% of the within mining sample population (Table 2). This could, perhaps, be due to males in the household being more likely to work outside of the home, resulting in increased exposure to iHg in the atmosphere. Individuals with occupations in mining or professional/urban occupations had 0.13- and 0.25-times lower odds of having hair %MeHg < 66% than unemployed individuals, respectively. The mining process consists of many steps, not all of which involve direct work with Hg^0^_L_, which may explain the miners with lower odds of exposure [65,66]. Additionally, many miners operate illegally [67], so it is possible that individuals would not accurately report their occupation on the survey.

### 3.4. Hair THg as a Predictor of Hair MeHg Exposure

**Figure 3:**
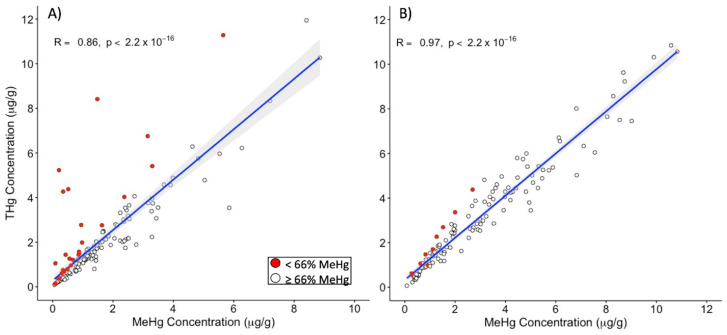
Scatterplots of Pearson correlations between hair THg and MeHg for individuals living within mining activity (**A**) and outside of mining activity (**B**). The blue line indicates a linear trend with the 95% confidence interval shaded in grey. Individuals with hair %MeHg < 66% are highlighted in red.

The observation of subgroups with mixed mercury exposure raises a question about the utility of hair THg as a population biomonitoring tool for MeHg exposure for mining communities. To answer this question, we first tested for correlation between hair THg and MeHg contents for mining and non-mining communities (Table S11, Figure 3). High correlations between hair THg and MeHg would indicate hair THg to be consistent with MeHg levels in hair (and MeHg brain burdens). We hypothesized that hair MeHg-THg correlations would be high (r > 0.7) in native communities and in communities outside of mining due to individuals primarily being exposed to MeHg via diet. Conversely, MeHg-THg correlations were hypothesized to be low (r < 0.5) in communities within mining due to multiple Hg species exposures (i.e., some of the total mercury would be present as iHg). Contrary to this hypothesis, significant positive correlations (r > 0.7) were found for populations both within and outside of mining, as well as for native and non-native communities (Figure 3). Moreover, the correlation coefficients for the within and outside of mining groups were not significantly different from one another (*p* = 0.261), indicating that the relationship between hair THg and hair MeHg were the same in mining and non-mining towns. Further examination of each community also showed significantly high correlations for 15 of 16 communities. The sample size for four of the communities was insufficient for this analysis. These results were in agreement with previous reports of strong hair MeHg-THg correlations in communities near mining activity in Brazil [68].

### 3.5. Subsample Selection Bias and Comparisons

This study entailed a selected subsample (N = 287) of the parent cohort study (N = 1543), and demographics of the subsample are comparable to the larger cohort. Both populations exhibited similar distributions with respect to sex, age, residence within mining, nativity, and occupation (Table S6). Individuals not selected from the parent cohort into the subsample did not significantly differ by THg exposure (t = 0.170, df = 605.9, *p* = 0.865) or sex (χ^2^ = 0.236, *p* = 0.627), further suggesting that the subsample likely does not have selection bias.

Our results also suggest that the exposure trends in the subset selected for this study are comparable to those of the larger cohort study, which found residence in a native community as a significant factor associated with THg in hair, predicting 1.9 times higher hair THg levels than non-native communities [53]. In this study, we similarly found that residence in a native community predicted 2.18 (95% CI: 1.38–3.35) times higher hair THg levels than non-native communities (Table S7). In both studies, residence within/outside of mining, age, and occupation were not significantly associated to hair THg at the 0.05 level (Table S7, Figure S8). In this sub-study, however, sex was a significant factor associated with THg in hair, predicting 24% lower hair THg levels in females compared to males (95% CI: 6.3–29.3%).

## 4. Discussion

This study demonstrates that the use of hair total mercury content is a valid biomarker for public health monitoring of MeHg exposure in both ASGM and non-ASGM communities. While total hair mercury alone is not sufficient to capture all forms of mercury exposure, this research shows that excluding hair THg assessments without an alternative biomarker for MeHg exposure could lead to significant underestimation of population exposure risks. As such, this study could alleviate uncertainty concerns for the use of hair THg for community biomonitoring of MeHg exposure in ASGM areas [1,58,59].

Here, we observed approximately 20% of the individuals residing within mining to have low hair %MeHg. Because these individuals represented a small percentage of the population, they did not alter population level trends for relative MeHg content in hair. Furthermore, we observed a nominal contribution of community location relative to mining activity as a predictor for %MeHg values and also observed strong hair MeHg-THg correlations for populations near mining. Altogether, these data support the use of hair THg as a population monitoring tool for MeHg exposure for Madre de Dios communities near mining activity.

Our conclusion differs from previous studies, suggesting that the hair THg biomarker overestimates MeHg exposure for individuals living near ASGM activity [21,22,24,69]. The discrepancy between our study and others exists for several reasons. First, the previous studies relied on small convenience samples (i.e., purposefully sampled individuals) of predominantly miners from the same communities, indicating that the study groups represented only a fraction of those at risk to mercury exposure and are not generalizable to the communities in which they live. Second, in our study, all communities reside within the same watershed, and regardless of mining activity, rely on the same local fish populations for dietary protein. As a result, for communities within the same watershed, hair THg correlates with MeHg exposure at the community level, regardless of nativity or location relative to mining.

While our results support the use of hair THg for public health monitoring of MeHg exposure in ASGM-impacted regions, the small subset of individuals with low %MeHg confirms exposure to multiple Hg sources (i.e., inorganic and organic) in the mining communities. As such, we recommend coupling THg hair with a companion biomarker for iHg exposure (i.e., urine [70]) for both organic and inorganic mercury exposure monitoring.

The implications of this study are relevant due to the implementation of the Minamata Convention and related efforts to improve public health assessments of mercury exposure. Several articles of the Convention emphasize development of methods to identify at-risk populations, as well as to evaluate the long-term effectiveness of the Convention in terms of reducing mercury exposure [23,71]. Population biomonitoring is therefore needed to understand mercury exposure trends [72], and governmental agencies of signatory nations are currently evaluating options and updating their policies for assessing mercury exposures in their populations [73]. Such polices for populations living near ASGM activity can be especially challenging because of the risks of exposure to multiple Hg species. International public health guidelines [1,4,58,59] for mercury biomonitoring currently recommend hair THg for populations where dietary MeHg is the major route of mercury exposure. Hair is advantageous over other biospecimens because it represents a time-integrated exposure, can be non-invasively collected, and is relatively easy to handle and analyze (compared to liquid specimens). However, these same international public health guidelines also express caution for the use of the hair THg biomarker in the ASGM context [1,4,60,61]. Such cautionary messaging may be discouraging the collection of the hair THg biomarker in ASGM regions without the replacement of alternative measures such as MeHg in hair or blood that require specialized equipment and technical training and may not be feasible in cost-constrained areas.

Such biomonitoring practices that exclude hair THg analyses without another biomarker of MeHg exposure are contributing to a scarcity of MeHg exposure data in regions hosting ASGM activity and possibly an overreliance on urine THg for identification of populations vulnerable to mercury exposure. In our study, 106 out of 150 individuals living in mining communities had hair THg > 1 μg/g (the EPA reference level), and 88 of these people had %MeHg > 66% for their hair. According to our data, sole reliance of urine THg for monitoring mercury exposure in mining communities would have misclassified approximately 59% of the population (i.e., 88 out of 150) as having low mercury exposure. This misclassification, combined with MeHg exposure having deleterious health effects, would significantly underestimate population exposure risks.

## 5. Conclusions

This study supports the use of hair THg for monitoring MeHg exposure of populations in mining regions where alternative MeHg exposure biomarkers are not feasible. We emphasize that our study does not advocate the sole use of hair THg as a biomarker of total mercury exposure (organic + inorganic Hg) in population biomonitoring. Rather, the collection of hair THg could supplement urine THg, the current recommended biomarker for communities near ASGM [3,4], and would provide a fuller picture of MeHg and iHg exposures in the community. We also note that for a subset of individuals in this study, hair THg also exhibited changes over distal hair segments, indicating changes in mercury exposures over time. Thus, temporal variability should be taken into consideration when interpreting exposure data. Overall, these results support the use of hair as an acceptable matrix for assessing community MeHg exposure near ASGM sites and underscore the importance of population-representative studies for use in biomonitoring programs.

## Figures and Tables

**Table 1:** Descriptive statistics for hair MeHg, hair THg, and %MeHg for participants within versus outside of mining as well as in native versus non-native communities along the MDD and Puquiri rivers. Communities outside of mining: Salvacion, Shintuya *, Shipitiari *, Diamante *, Isla de los Valles *, Boca Manu, Puerto Azul *, San Lorenzo *, Quincemil. Communities within mining: Masenawa *, Boca Isiriwe *, Boca Colorado, Puerto Luz *, Puquiri, Choque, Huepethue, Querada Nueva, Caychihue, Punquiri. *Native Community.

Location	n	Males	Females	Age (Years)	MeHg (μg/g)			THg (μg/g)			%MeHg			Number of Individuals with %MeHg > 66% (Percentage of n)
					Min.	Max.	Avg. ± s.d.	Min.	Max.	Avg. ± s.d.	Min.	Max.	Avg. ± s.d.	
**Outside Mining**	137	38	99	35.8 ± 10.4	0.080	10.8	2.87 ± 2.45 *	0.070	10.8	3.04 ± 2.37 *	16.0	145	91.3 ± 20.0 *	127 (92.7%)
**Within Mining**	150	46	104	36.2 ± 10.9	0.059	8.85	1.64 ± 1.58	0.090	11.9	2.14 ± 2.08	4.00	171	81.0 ± 25.9	121 (80.7%)
**Native**	88	28	60	33.6 ± 8.96	0.45	10.8	3.92 ± 2.41 *	0.403	11.3	4.15 ± 2.38 *	34.0	145	93.4 ± 20.2 *	81 (92.0%)
**Non-Native**	199	56	143	37.0 ± 11.1	0.059	8.85	1.47 ± 1.47	0.070	11.9	1.87 ± 1.83	4.00	171	82.6 ± 24.7	167 (83.9)
**Overall**	287	84	203	36.0 ± 10.6	0.059	10.8	2.22 ± 2.13	0.07	11.9	2.56 ± 2.27	4.00	171	86.0 ± 23.9	248 (86.4%)

* Denotes statistically significant difference (*p* < 0.001) between groups (i.e., outside vs. within mining, and native vs. non-native).

**Table 2:** Demographics of individuals within mining, separated by those with hair %MeHg < 66% and those with hair %MeHg ≥ 66%. Sex, age, nativity, and occupation were used as predictor variables in the logistic regressions.

Variable	%MeHg < 66% (N = 29)		%MeHg ≥ 66% (N = 121)	
	N	%	N	%
**Sex**				
Male	12	41	34	28
Female	17	59	87	72
**Age**				
<31	10	34	43	36
31–50	14	48	51	42
>50	5	18	27	22
**Native Ethnicity**				
Yes	2	7	15	12
No	27	93	106	88
**Occupation**				
Mining	1	3	12	10
Agriculture/Fishing	2	7	4	3
Other Outdoor	1	3	4	3
Professional/Urban	6	21	41	34
Self-employed/Other	5	18	22	18
No Job/Not Reported	14	48	38	32
**Length of Residence**				
Born Here	4	14	19	16
≤5 years	4	14	20	17
>5 years	16	54	73	60
NA	5	18	9	7

## Data Availability

The data presented in this study are available on request from the corresponding author.

## Supplementary Materials

The following Supplementary Information (SI) is available online at https://www.mdpi.com/article/10.3390/ijerph182413350/s1, SI Section S1: Hair Hg biomarker data from previous reports; SI Section S2: MeHg analysis methods; SI Section S3: Measurement uncertainties; SI Section S4: Hair Segment Data; SI Section S5: Statistical Analyses.
