# Supplementary material for: Efficacy of Hair Total Mercury Content as a Biomarker of Methylmercury Exposure to Communities in the Area of Artisanal and Small-Scale Gold Mining in Madre de Dios, Peru
Source: Int J Environ Res Public Health. 2021 Dec 18;18(24):13350. doi: 10.3390/ijerph182413350 (PMC8707462; doi:10.3390/ijerph182413350)
Supplement: Supplementary file 1 [file ijerph-18-13350-s001.zip › ijerph-1470088-supplementary.pdf]

Supplementary Information for:

Efficacy of hair total mercury content as a biomarker of methylmercury exposure to communities in the area of artisanal and small-scale gold mining in Madre de Dios, Peru

Faye Koenigsmark[1], Caren Weinhouse[2], Axel J. Berky[3], Ana Maria Morales[4], Ernesto Ortiz[5], Eric M. Pierce[6], William K. Pan[3,7*], Heileen Hsu-Kim[1*]

[1]Civil and Environmental Engineering, Box 90287, Duke University, Durham, North Carolina 27708, USA
[2]Oregon Health and Science University, 3181 SW Sam Jackson Park Road, Portland, Oregon, 97239, USA
[3]Nicholas School of the Environment, Duke University, 9 Circuit Drive, Durham, North Carolina, 27710, USA
[4]Centro Nacional de Salud Intercultural, Instituto Nacional de Salud, Ministerio de Salud, Cápac Yupanqui 1400 - Jesus María, Lima 11, Peru
[5]Duke Global Health Innovation Center, Duke University, 310 Blackwell Street, Durham, NC, 27701, USA
[6]Environmental Sciences Division, Oak Ridge National Laboratory, Oak Ridge, TN 37831, USA
[7]Duke Global Health Institute, Duke University, 310 Trent Drive, Durham, North Carolina 27710, USA

*Co-Corresponding authors:
William K. Pan: +1-919-684-4108, william.pan@duke.edu
Heileen Hsu-Kim: +1-919-660-5109, hsukim@duke.edu

Table of Contents

Section S1. Summary of Previously Published Data

Table S1. Summary of previous studies measuring total mercury (THg) and methylmercury (MeHg) in hair samples from individuals near/within ASGM activity S3

Table S2. Summary of studies reporting average (avg.) and standard deviation (s.d.) of %MeHg in hair for individuals from fish-consuming populations S4

Section S2: Reference Material and Internal Standard Recoveries for THg and MeHg Analyses

Figure S1. Histogram of recoveries of the THg Certified Reference Material (CRM) S5

MeHg Analysis Method for Hair Samples and Internal Standard Recoveries S5

Figure S2. Histograms of $CH_3{}^{201}HgCl$ internal standard recovery in MeHg Hair Reference Material (RM) and the resulting MeHg RM recovery...S6

Figure S3. Relationship between recovery of $CH_3{}^{201}HgCl$ internal standard in MeHg RMs and the resulting recovery of the RM itself S6

Figure S4. Histogram of $CH_3{}^{201}HgCl$ internal standard recoveries in proximal and temporal hair samples and the relationship with MeHg and %MeHg measurements S7

Figure S5. Comparison of hair MeHg divided by THg contents (expressed as a percentage) for study cohort when the $CH_3{}^{201}HgCl$ internal standard recovery cutoff is 40% and 70% S8

Section S3: Measurement Uncertainty Analysis for Hair THg and MeHg

Hair Total Mercury Uncertainty S9

Table S3. Determination of biological variability of THg via triplicate measurement of hair total mercury S10

Table S4. Summary of relative standard uncertainty for each component of the hair THg measurements..S11

Hair Total Methylmercury Uncertainty S11

Table S5. Summary of relative standard uncertainty for each component of the hair MeHg measurements. S12

Section S4. Hair Segment Analysis

Figure S6. %MeHg results for 2-cm hair segments of individuals in Diamante and Huepetuhe........S14

Figure S7. Total mercury and methylmercury results for 2-cm hair segments of individuals in Diamante and Huepetuhe..S15

Section S5. Statistical Analyses: Predictors of Hair THg and MeHg content

Table S6. Description of predictor variables used in mixed effect models S16

Table S7. Mixed effects models showing determinants of hair THg levels S17

Figure S8. Forest plots of determinants of hair THg levels S17

Table S8. Mixed effects models showing determinants of hair MeHg levels S18

Figure S9. Forest plots of determinants of hair methylmercury levels S18

Table S9. Mixed effects models showing determinants of hair %MeHg level...........................S19

Figure S10. Forest plots of determinants of hair %MeHg levels S19

Table S10. Odds ratios of individuals within mining having %MeHg < 66%...............................S20

Figure S11. Forest plots of determinants of hair %MeHg < or $\geq$ 66%....................................S20

Figure S12. Predicted probabilities of hair %MeHg <66% by job and sex...............................S21

Table S11. Pearson correlations between natural log (ln) transformed hair THg and MeHg contents by community groupings S22

Section S6: SI References..S23

Section S1. Summary of Previously Published Data

Table S1. Summary of previous studies measuring total mercury (THg) and methylmercury (MeHg) in hair samples from individuals near/within ASGM activity.

Authors	Study Region	Sample Size	THg Range (μg/g)	MeHg Range (μg/g)	%MeHg Range	Cohort Description
Sherman 2015[1]	Kegitia, Ghana	7	0.61-1.71	0.06-0.20	8-29	Gold miners
	Sekotong, Indonesia	4	2.75-5.49	1.52-2.15	32-55, 72	3 gold miners, 1 farmer
Laffont 2011[2]	Bolivia	5	0.17-2.35	0.12-0.46	9-40	Native gold miners
		17	0.23-2.25	0.073-0.99	4-86	Alluvial gold miners
Harada 1999[3]	Simbasirori, Tanzania	3	5.95-94.3	0.30-1.22	1.0-20.5	Gold miners
	Seweya, Tanzania	3	48.2-416	0.62-1.52	0.2-1.7	Fishing village near ASGM
	Mwanza, Tanzania	3	80.2-474	3.42-5.3	1.1-4.7	City dwellers near ASGM
Akagi 1995[4]	Itaituba, Brazil	7	0.5-110[a]	0.3-3.0[a]	1.5-50[b]	Gold miners
		4	7.5-32.3[a]	0.95-3.9[a]	6.7-53[b]	Gold shop workers
	Rainha, Brazil	11	2.4-21.4	1.9-29.4		
	Brasilia Legal, Brazil	37	3.5-46.9	0.9-42.6		
	Ponta de Pedras, Brazil	10	6.2-12.6	4.7-12.0	90[c]	Fishing village near ASGM
	Jacareacanga, Brazil	48	1.5-46.1	1.1-49.9		
	Tres Bocas, Brazil	11	8.4-53.8	6.1-50.3		
Ikingura 1996[5]	Mugusu, Tanzania	13	0.505-214	0.128-0.380	0.1-69.1	Inhabitants mining village
	Nungwe Bay, Tanzania	9	0.156-0.442	0.085-0.308	20.5-82.1	Fishing village downstream ASGM
Malm 1995[6]	Brasilia Legal, Brazil March 1992	28	2-46[a]	1-42[a]	4-110[b] (89[c])	Fish consuming, downstream mining
	August 1992	25	7-151	5-132	71-100 (85[c])	

[a]Estimated visually from reported figures; [b]calculated from estimated MeHg and THg values; [c]reported average

Table S2. Summary of studies reporting average (avg.) and standard deviation (s.d.) of %MeHg in hair for individuals from fish-consuming populations.

Reference	Country	N	% MeHg avg. ± s.d.
Gao *et al.* (2010)[7]	Belgium	10	71.2 ± 19.2
Kehrig *et al.* (1998) [8]	Brazil	20	95.0 ± 7.0
Dermelj *et al.* (1983)[9]	Greece, Yugoslavia	22	76.5 ± 29.0
Laffont *et al.* (2011)[10]	France	3	79.8 ± 17.6
Majed *et al.* (1999)[11]	Kuwait	100	95.6 ± 3.9
Majed *et al.* (1999)[11]	Kuwait	35	97.1 ± 2.5
Diez *et al.* (2009)[12]	Spain	65	75.0 ± 15.0
Diez *et al.* (2009)[12]	Spain	71	76.0 ± 17.0
Lebel *et al.* (1998)[13]	Brazil	91	89.6 ± 3.0
Soria *et al.* (1992)[14]	Spain	50	58.0 ± 26.0
Soria *et al.* (1992)[14]	Spain	17	90.0 ± 6.0
Akagi *et al.* (1995)[15]	Brazil	18	87.0 ± 5.0

Section S2: Reference Material and Internal Standard Recoveries for THg and MeHg Analyses

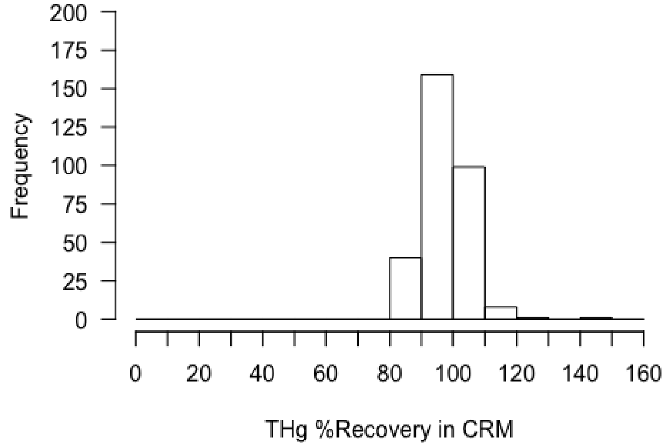

Figure S1. Histogram of the recoveries of the THg reference value for hair CRM (ERM-DB001) following analysis via thermal decomposition, amalgamation, and atomic absorption detection (Milestone DMA-80). The average recovery ± standard deviation was 98% ± 6.9% (n=308).

MeHg Analysis Method for Hair Samples and Internal Standard Recoveries

MeHg content was determined by extraction with tetramethylammonium hydroxide [16], followed by analysis of extracts by aqueous phase ethylation, purge-trap on Tenax resin and gas chromatographic separation (BrooksRand MerxM), and inductively coupled plasma mass spectrometry (Agilent 7700). Instruments were calibrated using dilutions of a Brooks Applied Labs certified 1 mg/L Methylmercury Standard. In order for the calibration curve to be used, each point on the curve had to be within 10% of the expected value. Additionally, calibration checks were performed every 15 to 20 samples during the batch run and also had to be within 10% of their expected value in order for subsequent measurements to be accepted.

With each batch of hair samples (approx. 20-30 samples), two samples of a hair reference material (RM) with a reference value of 0.258 μg/g (95% confidence interval 0.236 – 0.279 μg/g) (International Atomic Energy Agency 086) were processed in parallel for extraction and analysis. For all hair analyses, an aliquot of a stable MeHg isotope standard ($CH_3{}^{201}HgCl$) was added to each sample (RMs and hair samples) prior to the extraction step, and this MeHg spike was used as an internal standard (IS) to account for variable recoveries during the sample processing and analysis.

When accounting for IS recovery (shown in Figure S2A), the MeHg measurements of the RM averaged to 99% ± 8.4% (n=50) of the reference value (Figure S2B). The recovery of the $CH_3{}^{201}HgCl$ IS was not correlated with recovery of the reference MeHg value (Figure S3). As such, we established IS recovery of >40% as the threshold for inclusion of sample analyses in the reported results. For samples that met this threshold, the average IS recovery for proximal hair measurements (i.e., the data shown in Figure 2A) was 82% ±14% (Figure S4A). The average IS recovery for analysis of distal hair segments (i.e., the data shown in Figures S11 and S12) was 93% ±18% (Figure S4D). For both proximal and distal hair segments, MeHg content and %MeHg were not correlated with IS recovery (Figures S4B,C and S4E,F, respectively), indicating that variation in IS recovery was not greatly influencing MeHg results.

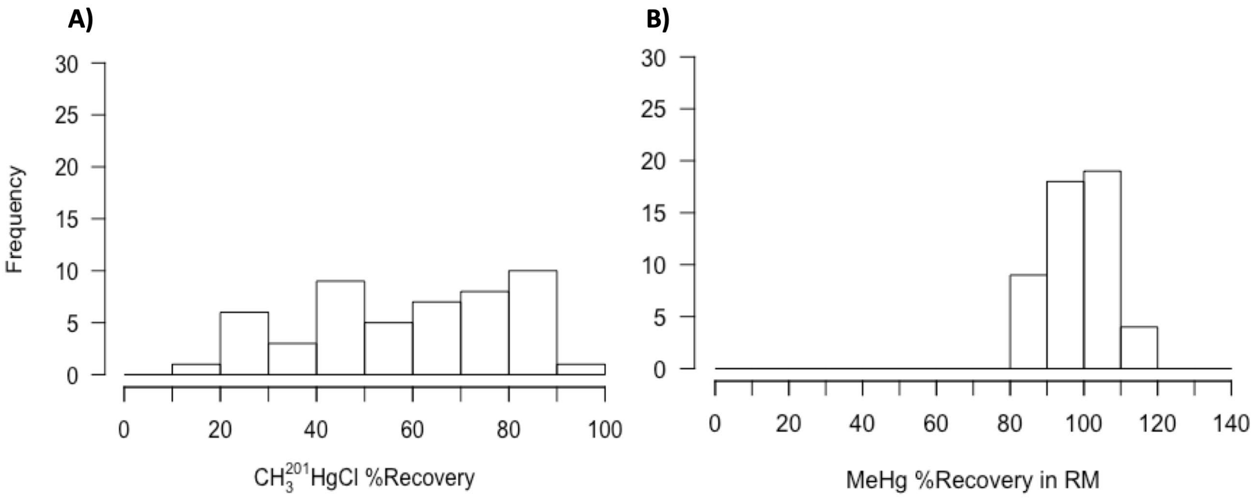

Figure S2. Histograms of (A) Recovery of the $CH_3{}^{201}HgCl$ internal standard spiked into the reference material (IAEA-086) for MeHg analysis; and (B) The measured recoveries of the published MeHg reference value for the reference material. The IAEA-086 RM was extracted and analyzed in duplicate with each batch of study samples, for a total of n=50 measurements of the reference material.

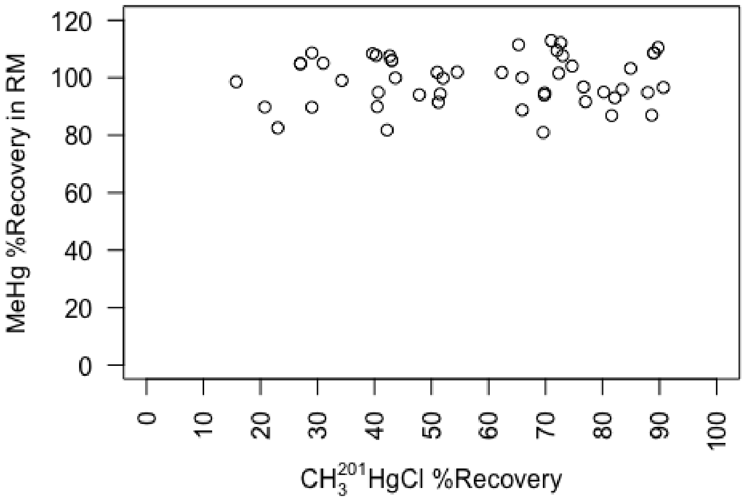

Figure S3. Relationship between recovery of the $CH_3{}^{201}HgCl$ internal standard and the % recovery of reference value for the RM. Despite internal standard recovery for the RM ranging from 10% to 100%, the measured MeHg in the RM was at least 80% of the reference value.

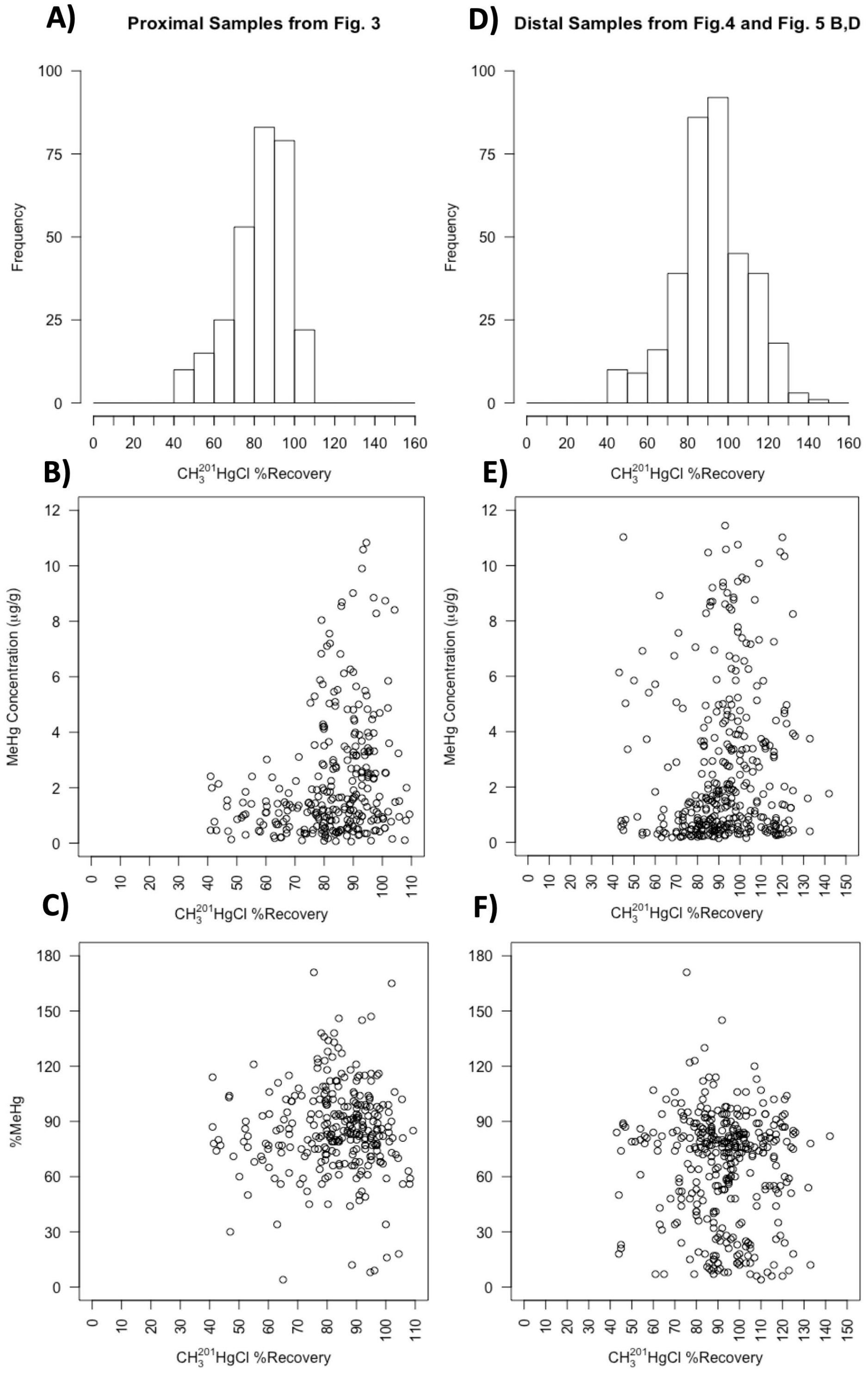

Figure S4. Histogram of %recovery of the $CH_3{}^{201}HgCl$ internal standard (IS) and relationships of this IS recovery to the measured hair MeHg contents and the percentage of the total Hg measured as MeHg for proximal hair segment samples (A-C) and distal hair segment samples (D-F).

In order to further ensure that MeHg IS recoveries were not influencing the results shown in Figure 2 of the text, we repeated our analysis with a more stringent minimum IS recovery of 70%. The change in the threshold removed 50 samples from our original analysis. This change in threshold also removed one community, Boca Isiriwe, as it only had N=1 sample in the original analysis. With this change, several key features were observed in a comparison of %MeHg plots (Figure S5). First, the outlier values (i.e. <20% and >120%MeHg) remained present with both minimum IS recovery thresholds, indicating that the outliers were not a result of low IS recovery during the analysis of these samples. Second, when the minimum IS recovery was changed from 40% to 70%, the same number of communities (16) have median %MeHg values within the expected %MeHg reference range (84% $\pm$ 18%; see Methods section of the main section of the publication). The expected %MeHg reference range is based on individuals whose only expected source of Hg exposure is dietary MeHg. Overall, these results suggest a lack of bias due to some sample analyses resulting is relatively low (40-70%) IS recovery. Regardless of the selected minimum value for IS recovery, the data show that hair THg was indicative of MeHg exposure for this population.

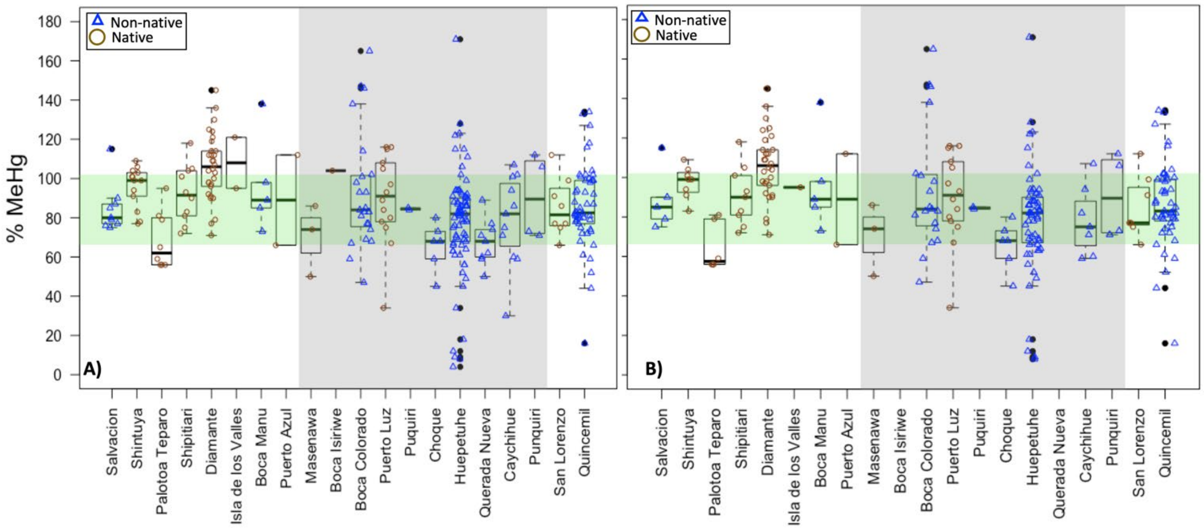

Figure S5. Measured hair MeHg divided by THg contents (expressed as a percentage) for each individual in the study cohort. (A) Analyses for which the recovery of the $CH_3^{201}HgCl$ internal standard was 40% or greater (same as Figure 3 in the main text; N= 287). (B) Analyses for which the recovery of the $CH_3^{201}HgCl$ internal standard was 70% or greater (N=237). Symbols represent measurements of individual participants and are grouped by their residence in native (open brown circles) and non-native (open blue triangles) communities. Communities in the grey shaded region lie within mining. The light green shaded region represents the expected range of %MeHg values, based on prior studies of Hg exposure for individual exposed to mercury only through diet. The black bars represent the median %MeHg value for each community, the box outline represents values in the 25th to 75th percentile, the whiskers represent values outside the middle 50%, and the closed black circles represent %MeHg values that are outliers of each community distribution. Data correspond to the proximal 2-cm hair segment.

Section S3: Measurement Uncertainty Analysis for Hair THg and MeHg

Measurement uncertainty associated with each parameter (THg and MeHg contents in hair) was evaluated based on methods described in previous guides[17–19]. Our evaluation first entailed the quantification of uncertainty in each component of the sample analysis (e.g., sample weights, liquid aliquots, reference material measurements) by performing replicate measurements for each component. From these repeated measurements, relative uncertainties were calculated and compared to each other as a mean to identify which steps in the analysis were dominant contributors to overall uncertainty of the measured THg and MeHg contents.

Hair Total Mercury Uncertainty

Total mercury content in hair, $[THg]$,was calculated using the following equation:

$$[THg] = \frac{m_{THg}}{m_{hair}} \quad \text{(S1)}$$

where m_{THg} is the mass of THg measured for a hair sample with mass m_{hair}. The overall uncertainty of the [*THg*] value depended on uncertainties related to m_{THg} (i.e., instrument calibration), m_{hair}, as well as measurement bias (*B*), measurement imprecision as determined by measured THg values of certified reference material ($[THg]_{CRM}$), and biological variability of hair THg levels across the scalp (*bio*). The standard uncertainty *u*(*x*) for each parameter *x,* was calculated as a relative term, as described below:

$u(m_{THg})/m_{THg}$: The instrument signal was calibrated using aliquots of a dissolved Hg(II) standard solution, and the calibration curve was determined by least squares regression of 5 - 7 calibration points. The instrument was calibrated periodically for samples in this study. The uncertainty of the measured m_{THg} was based on average relative residual error over all calibration points (*n*) that were used to determine calibration curves:

$$\frac{u(m_{THg})}{m_{THg}} = \frac{1}{n}\left(\sum_{i=1}^{n} \frac{m_{THg,i}^{E} - m_{THg,i}^{P}}{m_{THg,i}^{E}}\right) \quad \text{(S2)}$$

where the residual is the difference between the expected Hg mass $m_{THg,i}^{E}$ for calibration standard i and the Hg mass $m_{THg,i}^{P}$ predicted by the respective calibration curve.

$u(m_{hair})/m_{hair}$: Prior to analysis for Hg content, the mass of hair specimen m_{hair} was quantified on a standard precision balance (Mettler Toledo AG245). The relative uncertainty associated with the mass measurement $u(m_{hair})/m_{hair}$was defined as the relative standard deviation (RSD) of measured mass values for six aliquots of water (0.1 mL or 100 mg) dispensed on a weigh boat on the balance.

$u(bio)/\overline{[THg]}$: For 27 individuals, THg hair measurements were performed for hair tufts collected from 3 different parts of the scalp for each individual. These triplicates were used to assess intraindividual (i.e., biological) variability for hair THg content. The RSD of the triplicate THg measurements was determined for each individual (Table S3). The relative uncertainty associated with biological variability of THg across the scalp, $\frac{u(bio)}{\overline{[THg]}}$, was defined as the average RSD for the 27 individuals ($\frac{u(bio)}{\overline{[THg]}}$=0.113 or 11.3%).

Table S3. Average hair total mercury concentrations and corresponding standard deviation (SD) and relative standard deviation (RSD) from measurement of 3 tufts of hair from an individual. The average RSD represents intraindividual variability in hair THg across the scalp. Data corresponds to the proximal 2-cm hair segment.

Individual ID	Community	Average Hg $\overline{[THg]}$ *µg/g*	SD *µg/g*	RSD
BC1	Boca Colorado	0.851	0.063	0.074
BC2	Boca Colorado	2.00	0.616	0.308
BC3	Boca Colorado	1.72	0.096	0.056
BM1	Boca Manu	16.84	0.397	0.024
CH1	Choque	0.625	0.037	0.059
DM1	Diamante	21.4	2.078	0.097
DM2	Diamante	19.4	1.346	0.069
DM3	Diamante	16.7	0.828	0.050
DM4	Diamante	21.3	1.910	0.090
HU1	Huepetuhe	1.02	0.006	0.006
HU2	Huepetuhe	0.319	0.032	0.099
HU3	Huepetuhe	0.848	0.620	0.732
HU4	Huepetuhe	5.794	1.01	0.174
HU5	Huepetuhe	15.9	0.538	0.034
HU6	Huepetuhe	14.6	3.10	0.212
HU7	Huepetuhe	0.574	0.014	0.024
HU8	Huepetuhe	13.3	0.042	0.003
HU9	Huepetuhe	16.7	2.30	0.138
HU10	Huepetuhe	13.0	0.579	0.044
HU11	Huepetuhe	20.5	2.30	0.112
HU12	Huepetuhe	0.765	0.120	0.157
PN1	Punquiri	0.750	0.022	0.030
PT1	Palotoa Teparo	2.30	0.729	0.318
QM1	Quincemil	1.14	0.021	0.018
SA1	Salvacion	0.490	0.027	0.054
SA2	Salvacion	0.347	0.004	0.012
SH1	Shintuya	2.72	0.143	0.052
		Average:		0.113

$u(imp)/\overline{[THg]_{CRM}}$: Uncertainty due to imprecision was calculated based on $[THg]_{CRM}$, the measured Hg content of the hair certified reference material (DB001, European Reference Materials) using the following equation:

$$u(imp) = \sqrt{u_{ref}^2 + u_{rep}^2} \quad \text{(S3)}$$

where u_{ref} is the standard uncertainty associated with the reference material (from the CRM certificate), and u_{rep} is the standard error of the mean of replicate (N=308) CRM measurements. The relative uncertainty was then calculated by normalizing the value for $u(imp)$ to the average THg measured in the CRM ($\overline{[THg]_{CRM}}$).

$u(B)/B$: Bias (*B*) refers to the systematic error of a measurement and can be assessed from repeated measurement of a known reference material[17, 18, 20, 21]. Here, we defined *B* as the average recovery of the measured THg relative to the certified THg value (0.365 mg/kg) for the hair CRM (averaged over N=308 separate measurements). *B*=100% recovery would indicate

no bias. The uncertainty of the bias $u(B)$ was calculated from the standard error of the mean of the THg CRM recoveries.

Altogether, the relative standard uncertainty for hair THg (Table S3) was calculated according to the following:

$$\frac{u([THg])}{[THg]} = k\sqrt{\left(\frac{u(m_{THg})}{m_{THg}}\right)^2 + \left(\frac{u(m_{hair})}{m_{hair}}\right)^2 + \left(\frac{u(bio)}{\overline{[THg]}}\right)^2 + \left(\frac{u(imp)}{\overline{[CRM]}}\right)^2 + \left(\frac{u(B)}{B}\right)^2} \quad \text{(S4)}$$

where $u([THg])$ is the standard uncertainty of the hair THg content ([*THg*]), and *k* is the coverage factor (*k*=1.96 to obtain a confidence level of 95%).

Table S4. Relative standard uncertainty for contributing components of the hair THg analysis.

	Calibration	Sample Weight	Biological	Precision	Bias	Total Relative Uncertainty (95% confidence level)
	$\frac{\boldsymbol{u(m_{THg})}}{\boldsymbol{m_{THg}}}$	$\frac{\boldsymbol{u(m_{hair})}}{\boldsymbol{m_{hair}}}$	$\frac{u(bio)}{\overline{[THg]}}$	$\frac{u(imp)}{[THg_{CRM}]}$	$\frac{\boldsymbol{u(B)}}{\boldsymbol{B}}$	$\frac{\boldsymbol{u([THg])}}{\boldsymbol{[THg]}}$
Relative uncertainty value (%)	2.96	0.443	11.3	3.94	0.402	24.2

Hair Methylmercury Uncertainty

The measured hair methylmercury content $[MeHg]$ (in µg/g) was calculated according to the following:

$$[MeHg] = \frac{m_{MeHg}}{m_{hair}} = \frac{\frac{m_{198}}{R_{201}A_{201}} \times \frac{\forall_T}{\forall_a}}{m_{hair}} \quad \text{(S5)}$$

where m_{MeHg} is the mass of MeHg measured in a digested hair specimen of mass m_{hair}. The mass for the $Me^{198}Hg$ isotope (m_{198}) was used for the sample MeHg and was adjusted based on the recovery R_{201} of the $Me^{201}Hg$ internal standard, the natural relative abundance of the ^{201}Hg isotope (A_{201}=0.1318), and corrections for aliquots of the extracts as represented by $\forall_T$ and $\forall_a$, the total sample volume and analyzed aliquot volume, respectively. Relative uncertainties for all of these parameters were evaluated, with the exception of A_{201} as it is a widely accepted value. These relative uncertainties included:

$u(m_{198})/m_{198}$ and $u(m_{201})/m_{201}$: Relative uncertainty of measured $Me^{198}Hg$ and $Me^{201}Hg$ was based on the average residual error of calibration points for the $Me^{198}Hg$ and $Me^{201}Hg$ calibration curves, as described in Equation S2.

$u(m_{hair})/m_{hair}$: The relative uncertainty associated with the mass of the hair specimen was calculated from the RSD of 8 repeated measures of a hair sample on the high precision microbalance (Mettler Toledo XP26) used for the hair samples.

$u(\forall_T)/\forall_T$ and $u(\forall_a)/\forall_a$: All volumes were determined gravimetrically. Therefore, the RSD was calculated from 6 repeated mass measurements each of a low (100 μL) and a high (1 mL) volume of water. This range corresponds to the order of magnitude of $\forall_a$ and $\forall_T$, respectively.

$u(imp)/\overline{[MeHg]_{RM}}$: Uncertainty due to imprecision was calculated as described for $u(imp)/\overline{[THg]_{CRM}}$, except with N=50 measurements of a MeHg hair reference material (RM) (International Atomic Energy Agency-086).

$u(B)/B$: Bias (B) was defined as the mean MeHg recovery for N=50 separate measurements of the RM - i.e., the measured $[MeHg]_{RM}$ relative to the certified $[MeHg]_{RM}$ value (0.258 μg/g). The uncertainty of the bias $u(B)$ was calculated from the standard error of the mean B.

Intraindividual variability of hair MeHg (i.e. biological variability) could not be included due to insufficient sample mass for replicate hair MeHg for each individual. Altogether, the relative standard uncertainty at 95% confidence level for the hair MeHg measurement, shown in Table S4 was calculated according to:

$$\frac{u([MeHg])}{[MeHg]} = k\sqrt{\left(\frac{u(m_{198})}{m_{198}}\right)^2 + \left(\frac{u(m_{201})}{m_{201}}\right)^2 + \left(\frac{u(m_{hair})}{m_{hair}}\right)^2 + \left(\frac{u(\forall_T)}{\forall_T}\right)^2 + \left(\frac{u(\forall_a)}{\forall_a}\right)^2 + \left(\frac{u(imp)}{\overline{[MeHg]_{RM}}}\right)^2 + \left(\frac{u(B)}{B}\right)^2} \quad \text{(S6)}$$

where $u([MeHg])$ is the total standard uncertainty of the measured hair MeHg content $[MeHg]$.

Table S5. Relative standard uncertainty for components of the hair MeHg analysis protocol.

	Calibration m_{198}	Calibration m_{201}	Sample Weight	Total Volume	Aliquot Volume	Precision	Bias	Total Relative Uncertainty (95% confidence level)
	$\frac{u(m_{198})}{m_{198}}$	$\frac{u(m_{201})}{m_{201}}$	$\frac{u(m_{hair})}{m_{hair}}$	$\frac{u(\forall_T)}{\forall_T}$	$\frac{u(\forall_a)}{\forall_a}$	$\frac{u(imp)}{\overline{[MeHg]_{RM}}}$	$\frac{u(B)}{B}$	$\frac{u([MeHg])}{[MeHg]}$
Relative uncertainty value (%)	4.85	2.80	0.122	0.299	0.443	4.37	1.20	14.2

As shown in Table S4 and S5, the relative uncertainty of the hair THg analysis was greater than the relative uncertainty of the MeHg. Note that all of the contributing uncertainties from the analysis protocol for both [*THg*] and [*MeHg*] were less than 5%. The exception was biological variability (i.e. intraindividual variability) for hair THg, with a relative uncertainty of 11.3%. Intraindividual variability was not available for the MeHg measurements. Altogether, this uncertainty analysis shows that intraindividual variation (i.e. biological variability in THg between tufts of hair) was the main contributor to overall uncertainty in the hair THg measurements.

Section S4: Hair Segment Analysis

Multiple individuals were found to have %MeHg values over 100% in proximal hair segments even though %MeHg values are theoretically bound between 0 and 100%. Note that these values are based on two different tufts of hair from the same individual and that intraindividual variability (e.g., Different hair follicle growth rates [2]) could contribute to variable levels of hair THg across the scalp. For example, in a subset of individuals (N= 27) from the parent study, triplicate hair samples from each individual were analyzed for THg. The relative standard deviation for THg of the three hair tufts was 11.3% (Table S3), which exceeds the relative standard uncertainty from all other components involved in measurement of THg and MeHg (SI Section 3; Table S4 and S5). (Triplicate samples for MeHg analysis were not available.) Furthermore, in our analysis of homogenized distal hair segments, the %MeHg values were all below 105% for the individuals tested (Figure S6).

For the N=8 individuals with %MeHg = 105% to 170%in the proximal non-homogenized segment, the adjacent (homogenized) distal segment had %MeHg values within the reference range (Figure S6, red markers). Seven of these 8 individuals did not recently travel outside the community, which could change the %MeHg levels. Thus, the change in %MeHg levels between the proximal and distal segments was likely due to analysis with a homogenized sample.

For the N=6 individuals with %MeHg < 66% in proximal hair segments, the %MeHg value remained below the reference range for %MeHg in the second segment for 5 of these individuals (Figure S6, yellow markers). For individuals with proximal %MeHg between 66%-102%, 8 of 9 individuals remained within the %MeHg reference range for %MeHg in the second segment (Figure S6, green markers). This consistency between the proximal and second segment suggests that the proximal %MeHg <102% in Figure 2C are not explained by intraindividual variability, contrary to observations of proximal %MeHg values greater than 102%. These results highlight the value in utilizing splits of homogenized hair sample for analysis of multiple analytes.

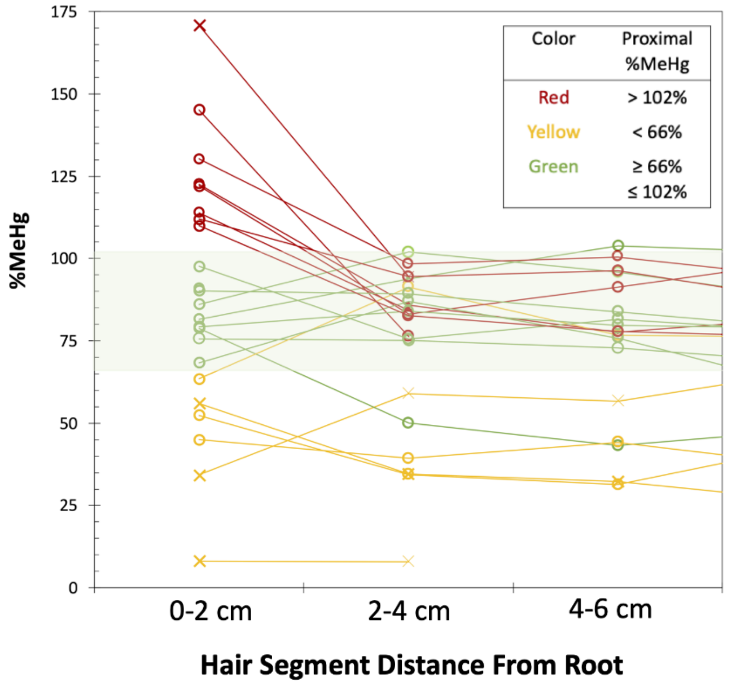

Figure S6. %MeHg values for 2-cm hair segments for individuals from 2 communities: Diamante, a native non-mining community (N=15) and) Huepetuhe, an urban mining community (N =8). Each line represents 1 female participant in the study, and each symbol is the %MeHg of a 2-cm segment, assumed to represent 2 months of hair growth. The light green shaded region represents the expected range of %MeHg values (66%-102%), based on prior studies of Hg exposure for individual exposed to mercury only through diet. Participants are therefore designated into one of the following groups based on their proximal %MeHg value: %MeHg < 66% (yellow; N = 6); 66% ≤ %MeHg ≤ 102% (green; N = 9); %MeHg > 102% (red; N = 8). Samples at 0-2cm correspond to the proximal segment (i.e. closest to the scalp) and different hair tufts for THg and MeHg analyses. Samples at 2-4cm correspond to two hair tufts that were homogenized and split for THg and MeHg analysis. Individuals who are in the non-migration cohort are denoted with the circle (○) symbol.

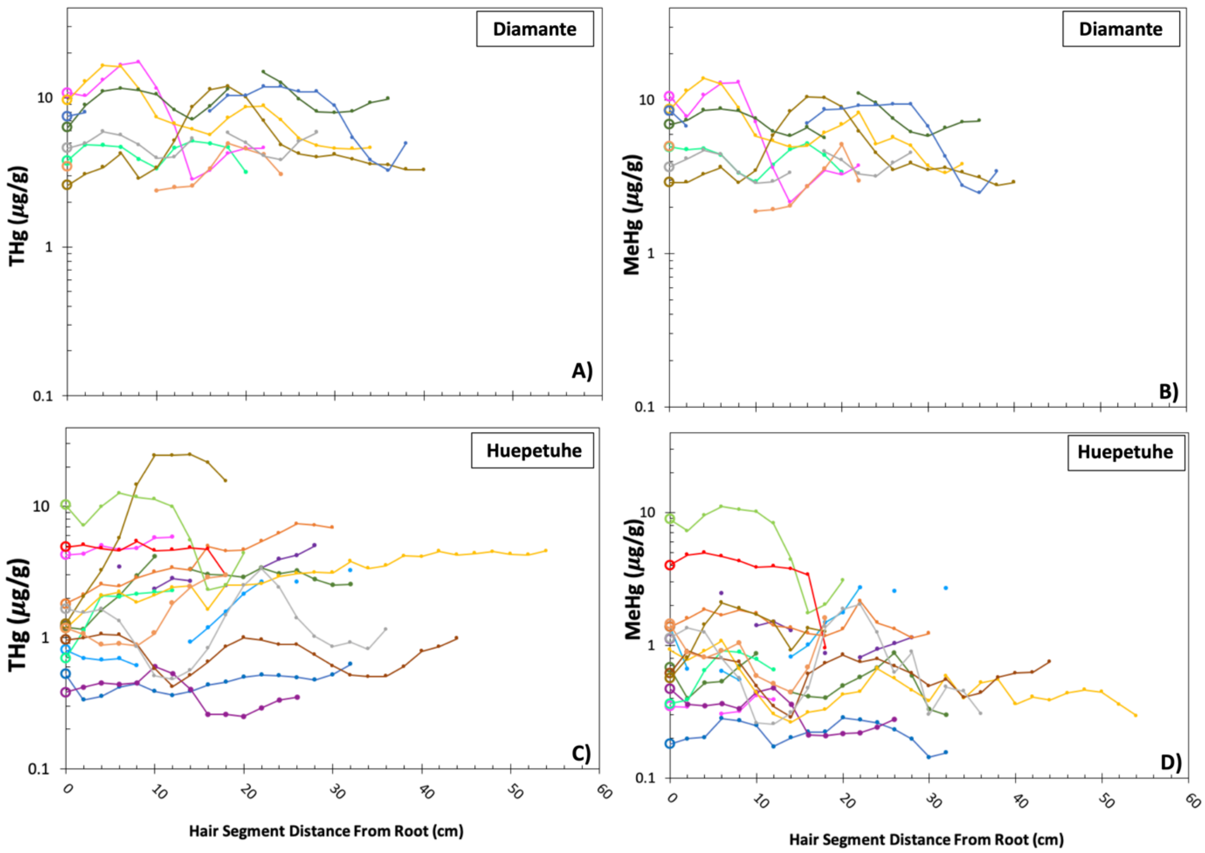

Figure S7. Total mercury and methylmercury contents for 2-cm hair segments from individuals living in: A, B) Diamante, a native non-mining community; and C,D) Huepetuhe, an urban mining community. Each line represents 1 female participant in the study, and each symbol is the THg or MeHg of a 2-cm segment, assumed to represent 2 months of hair growth. Open circles (at 0-2 cm) correspond to the proximal segment (i.e. closest to the scalp) and samples that were not homogenized and split prior to THg and MeHg analysis. Closed circles correspond to composite hair samples that were homogenized and split for THg and MeHg analysis. Gaps in line are due to measurements that could not be completed due to inadequate sample mass.

Section S5. Statistical Analyses: Predictors of Hair THg and MeHg content

Table S6. Description of predictor variables used in mixed effect models.

Variable	Subsample (N=287)		Population[22] (N = 1543)	
	N	%	N	%
**Sex**				
Male	84	29	473	31
Female	203	71	1070	69
**Age**				
<31	106	37	692	45
31-50	124	43	576	37
> 50	57	20	275	18
**Within Mining**				
Yes	150	52	797	52
No	137	48	746	48
**Nativity**				
Yes	88	31	358	23
No	199	69	1185	77
**Occupation**				
Mining	14	5	72	5
Agriculture/Fishing	31	11	141	9
Other Outdoor	12	4	49	3
Professional/Urban	81	28	339	22
Self-employed/Other	42	15	265	17
No Job/Not Reported	107	37	677	44

Table S7. Mixed effects models showing determinants of hair total mercury levels. Mixed effects models entailed natural log-transformed hair THg content as the outcome variable and age, sex, residence in native community, occupation, and residence in a mining community as predictor variables, with random intercepts for community-level variance. To determine inclusion in a multivariate linear regression model, the bivariate association between each predictor variable and outcome hair THg were tested. Predictor variables were included in the multivariate analysis if the bivariate association tested significant (*P*<0.20). Abbreviations: coefficient estimate (***β***), confidence interval (CI), yes (Y).

	**ln (THg hair, μg/g) bivariate analysis**				**ln (THg hair, μg/g) multivariate analysis**			
	β	***P* Value**	**95% CI**		***β***	***P* Value**	**95% CI**	
**Intercept**	-------	--------	-------	--------	0.53	0.035	0.089	0.96
**Age (30-50)[1]**	0.24	0.021	0.038	0.45	0.20	0.057	-0.007	0.40
**Age (>50)[1]**	-0.13	0.34	-0.38	0.13	-0.21	0.13	-0.48	0.061
**Sex (Female)**	-0.20	0.058	-0.41	0.0056	-0.27	0.015	-0.49	-0.061
**Native (Y)**	0.84	0.0017	0.42	1.2	0.78	0.0065	0.32	1.21
**Within Mining (Y)**	-0.37	0.21	-0.92	0.19	-0.018	0.94	-0.46	0.42
Occupation[2]								
**Mining**	0.085	0.73	-0.40	0.59				
**Agriculture/Fishing**	-0.041	0.82	-0.39	0.31				
**Other Outdoor**	0.12	0.64	-0.37	0.61				
**Professional/Urban**	-0.081	0.51	-0.32	0.16				
**Own Job/Other**	0.024	0.87	-0.27	0.32				

[1]reference: Age <30 years [2]reference: unemployed

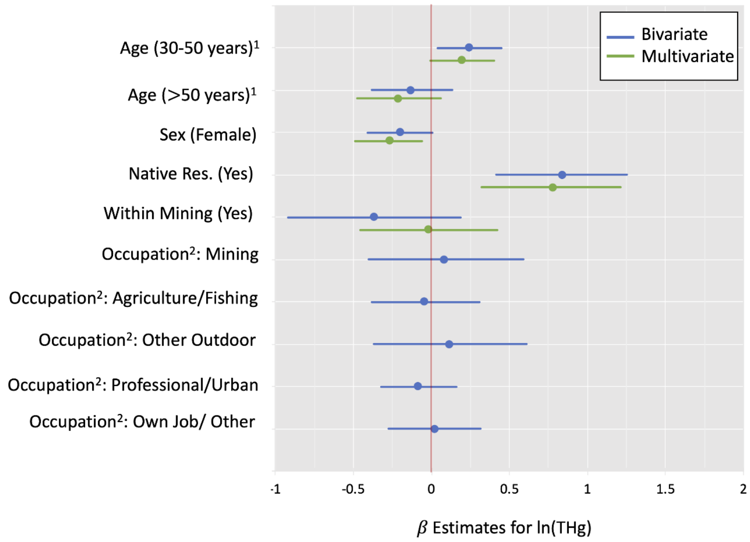

Figure S8. Determinants of hair total mercury levels shown as coefficient ***β*** estimates (circles) and 95% confidence interval (whiskers) for bivariate (blue) and multivariate (green) mixed effects models. [1]reference level: Age <30 years; [2]reference level: unemployed.

Table S8. Mixed effects models showing determinants of hair MeHg levels. The mixed effects models entailed natural log-transformed hair MeHg content as the outcome variable and age, sex, residence in native community, occupation, and residence in a mining community as predictor variables, with random intercepts for community-level variance. To determine inclusion in a multivariate linear regression model, the bivariate association between each predictor variable and outcome hair MeHg were tested. Predictor variables were included in the multivariate analysis if the bivariate association tested significant (P <0.20). Abbreviations : coefficient estimate (β), confidence interval (CI), yes (Y).

	ln (MeHg hair, μg/g), bivariate analysis				ln (MeHg hair, μg/g), multivariate analysis			
	β	P Value	95% CI		β	P Value	95% CI	
**Intercept**	-------	--------	--------	--------	0.20	0.46	-0.30	0.70
**Age (30-50)[1]**	0.25	0.018	0.044	0.46	0.25	0.020	0.039	0.45
**Age(>50)[1]**	-0.058	0.66	-0.32	0.20	-0.036	0.79	-0.29	0.23
**Sex (Female)**	-0.13	0.22	-0.34	0.077	-------	-------	-------	------
**Native (Y)**	0.88	0.0036	0.38	1.4	0.80	0.020	0.21	1.3
**Within Mining (Y)**	-0.50	0.12	-1.1	0.10	-0.18	0.56	-0.73	0.37
<u>Occupation[2]</u>								
**Mining**	0.26	0.30	-0.23	0.76				
**Agriculture/Fishing**	0.032	0.86	-0.31	0.38				
**Other Outdoor**	0.17	0.50	-0.32	0.66				
**Professional/Urban**	0.019	0.88	-0.22	0.26				
**Own Job/Other**	0.12	0.42	-0.18	0.41				

[1]reference: Age <30 years [2]reference: unemployed

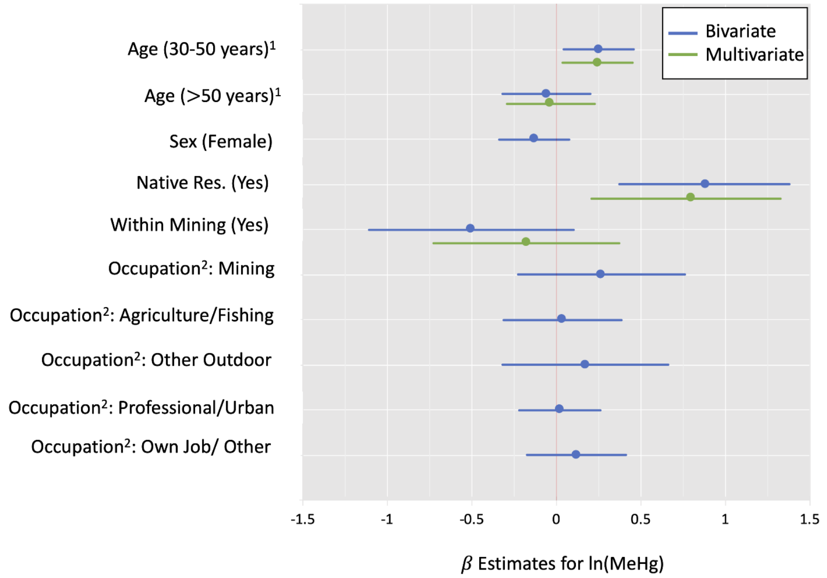

Figure S9. Determinants of hair methylmercury levels shown as coefficient β estimates (circles) and corresponding 95% confidence interval (whiskers) for bivariate (blue) and multivariate (green) mixed effects models. [1]reference level: Age <30 years; [2]reference level: unemployed.

Table S9. Mixed effects models showing determinants of hair %MeHg levels. The mixed effects models entailed the natural log-transformed hair %MeHg content as the outcome variable and age, sex, residence in native community, occupation, and residence in a mining community as predictor variables, with random intercepts for community-level variance. To determine inclusion in a multivariate linear regression model, the bivariate association between each predictor variable and outcome hair %MeHg were tested. Predictor variables were included in the multivariate analysis if the bivariate association tested significant (*P* <0.20). Abbreviations : coefficient estimate (***β***), confidence interval (CI), yes (Y).

	**ln (%MeHg hair) bivariate analysis**				**ln (%MeHg hair) multivariate**			
	β	***P* Value**	**95% CI**		***β***	***P* Value**	**95% CI**	
**Intercept**	-------	--------	-------	--------	4.4	2e-16	4.3	4.5
**Age (30-50)[1]**	0.026	0.62	-0.076	0.13	-------	--------	-------	--------
**Age(>50)[1]**	0.086	0.19	-0.042	0.21	-------	--------	-------	--------
**Sex (Female)**	0.048	0.35	-0.052	0.15	-------	--------	--------	--------
**Native (Y)**	0.11	0.13	-0.034	0.25	0.057	0.41	-0.095	0.21
**Within Mining (Y)**	-0.16	0.046	-0.27	-0.013	-0.14	0.12	-0.28	0.0012
Occupation[2]								
**Mining**	0.16	0.16	-0.062	0.39	0.19	0.11	-0.039	0.41
**Agriculture/Fishing**	0.098	0.24	-0.063	0.26	0.058	0.49	-0.11	0.22
**Other Outdoor**	0.072	0.55	-0.16	0.31	0.069	0.57	-0.16	0.31
**Professional/Urban**	0.094	0.11	-0.021	0.21	0.10	0.093	-0.014	0.21
**Own Job/Other**	0.029	0.69	-0.12	0.17	0.040	0.59	-0.11	0.18

[1]reference: Age <30 years [2]reference: unemployed

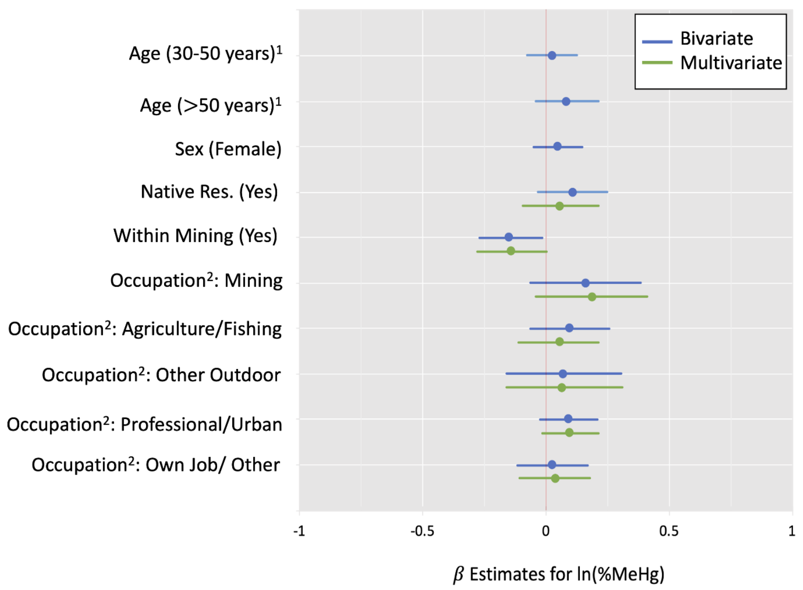

Figure S10. Determinants of hair %MeHg values shown as coefficient ***β*** estimates (circles) and corresponding 95% confidence interval (whiskers) for bivariate (blue) and multivariate (green) mixed effects models. [1]reference level: Age <30 years; [2]reference level: unemployed**.**

Table S10. Odds ratios (OR) for an individual within a mining community having hair %MeHg <66% based on age, sex, residence in native community, and occupation variables. Random intercepts were used to adjust for community-level variance. P-values are compared to an individual with hair %MeHg ≥66. To determine inclusion in a multivariate logistic model, the bivariate association between each predictor variable and outcome hair %MeHg were tested. Predictor variables were included in the multivariate analysis if the bivariate association tested significant (*P* <0.20). Abbreviations : confidence interval (CI), yes (Y).

	%MeHg <66 vs ≥66% bivariate analysis				%MeHg <66 vs ≥66% multivariate			
	OR	***P* Value**	**95% CI**		***OR***	***P* Value**	**95% CI**	
**Intercept**	-------	--------	-------	--------	1.3	0.64	0.41	4.2
**Age (30-50)[1]**	1.2	0.72	0.48	2.9	--------	--------	--------	--------
**Age(>50)[1]**	0.80	0.74	0.25	2.6	--------	--------	--------	--------
**Sex (Female)**	0.55	0.17	0.24	1.3	0.24	0.011	0.082	0.72
**Native Ethnicity (Y)**	0.52	0.41	0.11	2.4	--------	--------	--------	--------
Occupation[2]								
**Mining**	0.23	0.17	0.027	1.9	0.067	0.024	0.006	0.70
**Agriculture/Fishing**	1.4	0.74	0.22	8.2	0.56	0.57	0.074	4.2
**Other Outdoor**	0.68	0.74	0.070	6.6	0.19	0.19	0.016	2.3
**Professional/Urban**	0.40	0.086	0.14	1.1	0.25	0.022	0.077	0.82
**Own Job/Other**	0.62	0.41	0.20	1.9	0.43	0.19	0.13	1.5

[1]reference: Age <30 years [2]reference: unemployed

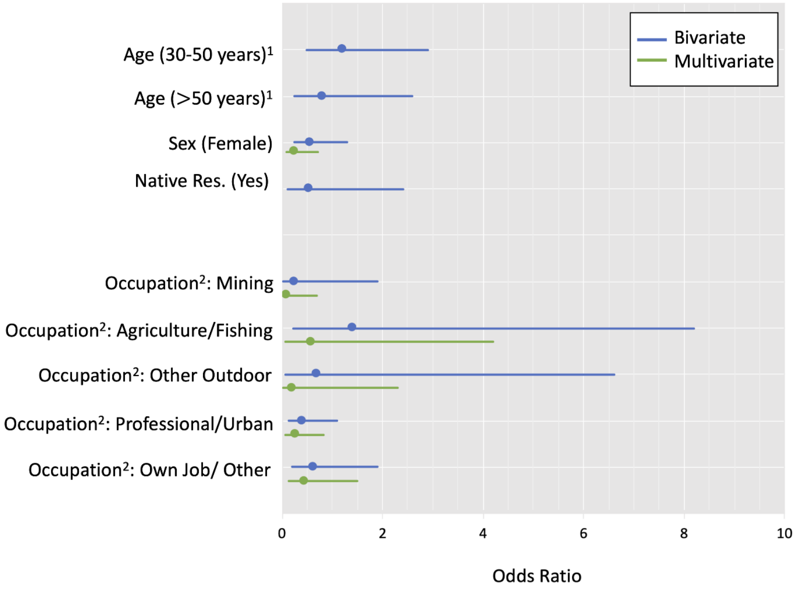

Figure S11. Odds ratio (circles) and 95% confidence interval (whiskers) for an individual within a mining community having hair %MeHg <66% based on predictor variables. Bivariate logistic regression results in are blue, and multivariate logistic regression models are in green. [1]reference level: Age <30 years; [2]reference level: unemployed**.**

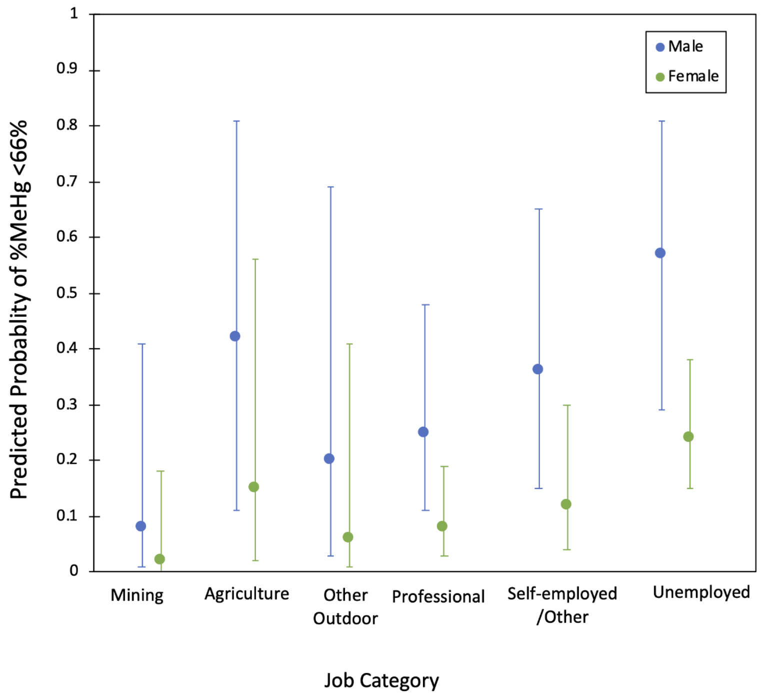

Figure S12. Predicted probabilities (circles) and 95% confidence interval (whiskers) of having hair %MeHg <66% by occupation and sex.

Table S11. Pearson correlations between natural log (ln) transformed hair total mercury and methylmercury contents by community groupings.

Community	N individuals	THg-MeHg Hair Correlations (r)
All	287	0.92
Within Mining	150	0.87
Outside Mining	137	0.97
Native	88	0.94
Non-native	199	0.89
Salvacion	9	0.97
Shintuya	13	0.98
Palotoa Teparo	8	0.90
Shipitiari	10	0.93
Diamante	30	0.93
Isla de los Valles	2	NA
Boca Manu	5	0.92
Puerto Azul	1	NA
Masenawa	3	0.43*
Boca Isiriwe	1	NA
Boca Colorado	23	0.89
Puerto Luz	14	0.75
Choque	6	0.97
Huepethue	77	0.83
Puquiri	2	NA
Querada Nueva	9	0.98
Caychihue	11	0.89
Punquiri	4	0.93
San Lorenzo	8	0.97
Quincemil	50	0.97

*not significant

Supplementary Information References

1. Sherman, L. S.; Blum, J. D.; Basu, N.; Rajaee, M.; Evers, D. C.; Buck, D. G.; Petrlik, J.; DiGangi, J. Assessment of Mercury Exposure among Small-Scale Gold Miners Using Mercury Stable Isotopes. *Environ. Res.* **2015**, *137*, pp. 226–234. https://doi.org/10.1016/j.envres.2014.12.021.
2. Laffont, L.; Sonke, J. E.; Maurice, L.; Monrroy, S. L.; Chincheros, J.; Amouroux, D.; Behra, P.; Monso, E.; Toulouse, F.-; Cedex, F.-P. A. U. Hg Speciation and Stable Isotope Signatures in Human Hair As a Tracer for Dietary and Occupational Exposure to Mercury. *Environ. Sci. Technol.* **2011**, *45*, pp. 9910–9916.
3. Harada, M.; Nakachi, S.; Cheu, T.; Hamada, H.; Ono, Y.; Tsuda, T.; Yanagida, K.; Kizaki, T.; Ohno, H. Monitoring of Mercury Pollution in Tanzania: Relation between Head Hair Mercury and Health. *Sci. Total Environ.* **1999**, *227*, 2–3, pp. 249–256. https://doi.org/10.1016/S0048-9697(99)00031-5.
4. Akagi, H.; Malm, O.; Branchesb, F. J. P.; Kinjoa, Y.; Kashima, Y.; Guimaraes, J.; Oliveira, R.; Haraguchi, K.; Pfeiffer, W.; Takizawa, Y.; Kato, H. Human Exposure to Mercury Due to Goldmining in the Tapajos River Basin, Amazon, Brazil: Speciation of Mercury in Human Hair, Blood, and Urine. *Water. Air. Soil Pollut.* **1995**, *80*, pp. 85–94.
5. Ikingura, J. R.; Akagib, H. Monitoring of Fish and Human Exposure to Mercury Due to Gold Mining in the Lake Victoria Goldfields , Tanzania. **1996**, *9697*, 96.
6. Malm, O.; Branchesb, F. J. P.; Akagi, H.; Castro, M.; Pfeiffer, W. C.; Haradac, M.; Bastosa, W. R.; Katob, H. Mercury and Methylmercury in Fish and Human Hair from the Tapajos River Basin, Brazil. *Sci. Total Environ.* **1995**, *175*, pp. 141–150.
7. Gao, Y.; De Galan, S.; De Brauwere, A.; Baeyens, W.; Leermakers, M. Mercury Speciation in Hair by Headspace Injection-Gas Chromatography-Atomic Fluorescence Spectrometry (Methylmercury) and Combustion-Atomic Absorption Spectrometry (Total Hg). *Talanta* **2010**, *82*, 5, pp. 1919–1923. https://doi.org/10.1016/j.talanta.2010.08.012.
8. Kehrig, A.; Malm, O.; Akagi, H.; Guimara, J. R. D.; Torres, P. M. Methylmercury in Fish and Hair Samples from the Balbina Reservoir , Brazilian Amazon. **1998**, *90*, 77, pp. 84–90.
9. Dermelj, M.; Horvat, M.; Byrne, A. R.; Stegnar, P. Mercury, Methyl-Mercury, and Selenium in Scalp Hair of Inhabitants from Mediterranean Areas. *Chemosphere* **1987**, *16*, 4, pp. 877–886.
10. Laffont, L.; Sonke, J. E.; Maurice, L.; Monrroy, S. L.; Chincheros, J.; Amouroux, D.; Behra, P. Hg Speciation and Stable Isotope Signatures in Human Hair as a Tracer for Dietary and Occupational Exposure to Mercury. *Environ. Sci. Technol.* **2011**, *45*, 23, pp. 9910–9916. https://doi.org/10.1021/es202353m.
11. Al-Majed, N. B.; Preston, M. R. Factors Influencing the Total Mercury and Methyl Mercury in the Hair of the Fishermen of Kuwait. *Environ. Pollut.* **2000**, *109*, 2, pp. 239–250. https://doi.org/10.1016/S0269-7491(99)00261-4.
12. Díez, S.; Delgado, S.; Aguilera, I.; Astray, J.; Pérez-Gómez, B.; Torrent, M.; Sunyer, J.; Bayona, J. M. Prenatal and Early Childhood Exposure to Mercury and Methylmercury in Spain, a High-Fish-Consumer Country. *Arch. Environ. Contam. Toxicol.* **2009**, *56*, 3, pp. 615–622. https://doi.org/10.1007/s00244-008-9213-7.
13. Lebel, J.; Mergler, D.; Branches, F.; Lucotte, M.; Amorim, M.; Larribe, F.; Dolbec, J. Neurotoxic Effects of Low-Level Methylmercury Contamination in the Amazonian Basin.

Environ. Res. **1998**, *79*, 1, pp. 20–32. https://doi.org/10.1006/enrs.1998.3846.

14. Soria, M. L.; Sanz, P.; Martínez, D.; López-Artíguez, M.; Garrido, R.; Grilo, A.; Repetto, M. Total Mercury and Methylmercury in Hair, Maternal and Umbilical Blood, and Placenta from Women in the Seville Area. *Bull. Environ. Contam. Toxicol.* **1992**, *48*, 4, pp. 494–501. https://doi.org/10.1007/BF00199063.
15. Akagi, H.; Malm, O.; Kinjo, Y.; Harada, M.; Branches, F. J. P.; Pfeiffer, W. C.; Kato, H. Methylmercury Pollution in the Amazon, Brazil. *Sci. Total Environ.* **1995**, *175*, 2, pp. 85–95. https://doi.org/10.1016/0048-9697(95)04905-3.
16. Laffont, L.; Maurice, L.; Amouroux, D.; Navarro, P.; Monperrus, M.; Sonke, J. E.; Behra, P. Mercury Speciation Analysis in Human Hair by Species Specific Isotope Dilution Using GC-ICP-MS. *Anal. Bioanal. Chem.* **2013**, *405*, pp. 3001–3010.
17. White, G. H. Basics of Estimating Measurement Uncertainty. *Clin. Biochem. Rev.* **2008**, *29 Suppl 1*, August, pp. S53-60.
18. Scheme, A.; Laboratories, F. O. R. T e c h n i c a l Gu i d e 2 A Guide on Measurement Uncertainty in Chemical & Microbiological Analysis. *Spring* **2008**, No. March.
19. Eurachem/CITAC. *Quantifying Uncertainty in Analytical Measurements*; 2012. https://doi.org/10.3329/jbas.v41i2.35494.
20. Allen, B. L.; Crawford, L. Guidance on Measurement Uncertainty for Medical Laboratories. **2014**, No. December, pp. 1–21.
21. Šimundić, A. M. Bias in Research. *Biochem. Medica* **2013**, *23*, 1, pp. 12–15. https://doi.org/10.11613/BM.2013.003.
22. Weinhouse, C.; Gallis, J. A.; Ortiz, E.; Berky, A. J.; Morales, A. M.; Diringer, S. E.; Harrington, J.; Bullins, P.; Rogers, L.; Hare-, J.; Hsu-kim, H.; Pan, W. K.; Estudios, C. De. A Population-Based Mercury Exposure Assessment near an Artisanal and Small Scale Gold Mining Site in the Peruvian Amazon. *JESEE* **2019**, pp. 1–19.
